# Origins, structures, and functions of circulating DNA in oncology

**DOI:** 10.1007/s10555-016-9629-x

**Published:** 2016-07-08

**Authors:** A. R. Thierry, S. El Messaoudi, P. B. Gahan, P. Anker, M. Stroun

**Affiliations:** 1 IRCM, Institut de Recherche en Cancérologie de Montpellier, INSERM U1194, F-34298 Montpellier, France; 2 135 route des fruitières, 74160 Beaumont, France; 3 6 Pedro-meylan, 1208 Geneva, Switzerland

**Keywords:** Cell-free circulating DNA, Cancer, Structures, Origins, Functions

## Abstract

While various clinical applications especially in oncology are now in progress such as diagnosis, prognosis, therapy monitoring, or patient follow-up, the determination of structural characteristics of cell-free circulating DNA (cirDNA) are still being researched. Nevertheless, some specific structures have been identified and cirDNA has been shown to be composed of many “kinds.” This structural description goes hand-in-hand with the mechanisms of its origins such as apoptosis, necrosis, active release, phagocytosis, and exocytose. There are multiple structural forms of cirDNA depending upon the mechanism of release: particulate structures (exosomes, microparticles, apoptotic bodies) or macromolecular structures (nucleosomes, virtosomes/proteolipidonucleic acid complexes, DNA traps, links with serum proteins or to the cell-free membrane parts). In addition, cirDNA concerns both nuclear and/or mitochondrial DNA with both species exhibiting different structural characteristics that potentially reveal different forms of biological stability or diagnostic significance. This review focuses on the origins, structures and functional aspects that are paradoxically less well described in the literature while numerous reviews are directed to the clinical application of cirDNA. Differentiation of the various structures and better knowledge of the fate of cirDNA would considerably expand the diagnostic power of cirDNA analysis especially with regard to the patient follow-up enlarging the scope of personalized medicine. A better understanding of the subsequent fate of cirDNA would also help in deciphering its functional aspects such as their capacity for either genometastasis or their pro-inflammatory and immunological effects.

## The origin of the cirDNA concept

### Extracellular DNA

The term extracellular DNA concerns both nuclear and/or mitochondrial DNA liberated from the cell. They are found in the physiological extracellular milieu, e.g., blood, lymph, bile, milk, urine, saliva, mucous suspension, spinal fluid, and amniotic fluid. These extracellular DNA molecules are found in humans and both the animal and plant kingdoms. In addition, extracellular DNA molecules are found and released in cell culture supernatants either from cell lines, primary cells, organoids, or embryo cultures [1].

### Extracellular circulating DNA

This term is reserved for extracellular DNA molecules found in the physiological circulating fluids (see Sect. 1.1). In the following work, these extracellular circulating nucleic acids will be designated by the term circulating DNA (cirDNA). We are specifically interested in the circulating DNA present in the blood.

### History

**Fig. 1:**
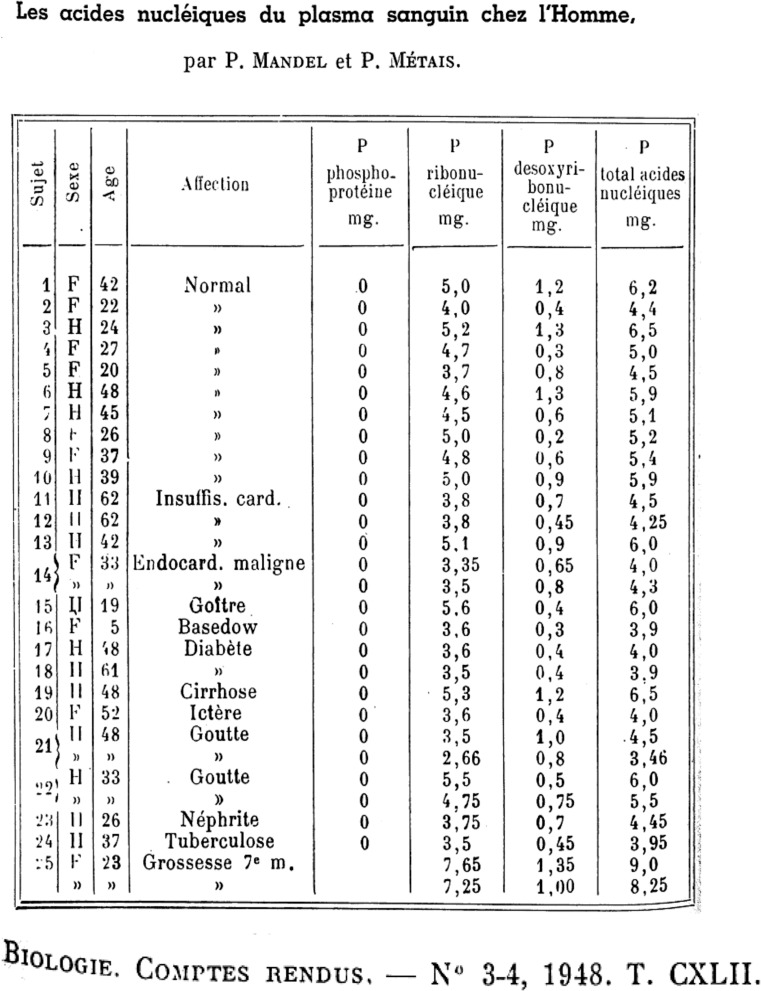
The first identification of extracellular nucleic acids in human blood compartment by Mandel and Metais in 1948 (adapted from Mandel and Métais [8])

Researchers in the 1940s and 1950s were very much geared to the determination of the chemical nature of the gene leading to the identification of DNA as the principle component and to the gene-DNA theory in 1957 (Table 1). As a consequence, the first demonstration of DNA and RNA in the blood of healthy individuals and diseased patients by Mendel and Métais in 1948 [8] was ignored (Fig. 1). Subsequent to their discovery, Tan et al., as late as 1966, showed that circulating DNA was present in the blood of systemic lupus erythematosus (SLE) patients leading to the formation of anti-dsDNA antibodies [27]. In the field of oncology, Leon et al. revealed, in 1977, that the concentration of cirDNA from cancer patients is greater than that from healthy individuals, opening the way to potential biomedical applications in oncology [21]. However, it was not until 1989 that cirDNA was recognized when Maurice Stroun and Philippe Anker showed that in cancer patients, cirDNA was, in part, of tumoral origin since it harbored the particular double-strand instability specific to tumor DNA [22]. In 1994, Vasioukhin et al. and Sorenson et al., in collaboration with P. Anker, found that cirDNA bore *RAS* point mutations specifically found in tumor cells [23, 24]. Since then, the concept of a “liquid biopsy” was born. In parallel, cirDNA had become of interest in another clinical domain: in 1997, Lo et al. showed that DNA of fetal origin circulated in the blood of pregnant women [25], permitting the early identification of fetal genetic anomalies, such as Down syndrome [28], through a simple maternal blood sample and to avoid amniocentesis and other invasive techniques that presented risks and complications. Analysis of fetal cirDNA from maternal blood collection additionally affords both sex and Rhesus factor determination [29, 30]. Concerning the field of medically assisted procreation, extracellular DNA analysis is promising: at the moment, pre-implantation diagnosis is made by aspiration of one or two cells from the embryo, imposing traumatic risks and consequences for the implantation of the embryos [31]. Extracellular DNA analysis from the embryonic culture medium will permit avoidance of these complications and yield genetic information by DNA sequence analysis and quality by its structure. Publication frequency for the past 50 years shows the increased interest by the community concerning cirDNA. Promising other potential clinical applications from cirDNA analysis were shown, such as for autoimmune diseases (SLE), inflammatory diseases (rheumatoid arthritis, Crohn’s disease), systemic disorders (granulomatosis with polyangiitis), trauma, sepsis, or myocardial infarction [32]. Since 2005, many clinical studies have been performed implicating a role for cirDNA; however, such analyses are still to be validated in clinical practice [32–35]. Table 1 provides a timeline of the main discoveries concerning cirDNA prior to the concept of “liquid biopsy” in oncology as well as those concerning the structural and functional aspects of cirDNA.

**Table 1:** Timeline for discoveries on cirDNA

Timeline discoveries on circulating cell-free DNA		
Date	Authors	Discovery
1871	Miescher	DNA isolation (nucleine) [3]
1929	Levene, Haller	Identification of first particular structures of DNA [4]
1941	Beadle, Tatum	One gene - one enzyme [5]
1944	McCLintock	Jumping genes [6]
1944	Avery et al.	DNA carrier of genetic information [7]
1948	Mandel, Métais	Circulating nucleic acids in human blood [8]
1949	Chayen, Norris	Cytoplasmic localization of DNA [9]
1950	Swift	DNA constancy [10]
1950	Chargaff	Base parity rule [11]
1953	Watson, Crick	Identification of DNA structure [12]
1953	Wilkins et al.	Identification of DNA structure [13]
1957	Sinsheimer	Gene concept [14]
1959	Gartler	DNA uptake by cells [15]
1962	Gahan, Stroun	DNA mobility [16, 17]
1965	Gahan, Chayen	Messenger DNA [18]
1972	Stroun, Anker	Active secretion of DNA by cells [19]
1977	Stroun et al.	Characterization and definition of cirDNA [20]
1977	Leon et al.	Higher cirDNA concentrations in cancer patients [21]
1989	Stroun et al.	Identification of tumor-derived cirDNA [22]
1994	Anker et al.	RAS mutation detection by cirDNA analysis [23, 24]
1997	Lo et al.	Identification of fetal derived cirDNA [25]
2005	Diehl et al.	Large cohorts of patients for screening of point mutations by cirDNA analysis [26]

Adapted from Gahan and Swaminathan [2]

A high proportion of the investigations on cirDNA directed to clinical relevance are in the field of oncology. The results obtained for many different cancers have opened a new research area indicating that plasma DNA might eventually be a suitable target for the development of non-invasive diagnostic, prognostic, treatment monitoring and follow-up tests for cancer [35].

## Biological aspects of cirDNA

### Origins

The determination of structural characteristics of cirDNA is still under investigation. Nevertheless, some structures have been identified and cirDNA has been shown to be composed of many “kinds”. This structural description goes hand-in-hand with the mechanisms of its origins; in effect, the cirDNA structures can be envisaged as signatures of the releasing mechanisms. While this research has progressed at the rhythm of discoveries concerning cirDNA, the technological revolution has permitted an increased sensitivity in the identification of these structures. The following section reconsiders the major discoveries since the 1960s.

#### General characteristics of circulating DNA

Since 1966, work on auto-immune pathologies has permitted the first characterization of cirDNA [27, 36–45]. These studies were based upon the constant finding that in SLE, dsDNA antibodies are found in the blood circulatory compartment. The hypothesis that the DNA could be found directly in the circulatory system complexed with the dsDNA antibodies was demonstrated and confirmed. Tan et al. were the first to show the presence of DNA in the human circulatory system of SLE patients [27]. This first discovery initiated a number of research studies in this area, leading to the first structural observations concerning cirDNA. Subsequently, the presence of a complex structure was shown to be composed of a variety of types of DNA of different sizes [45]. Some workers showed single-stranded fragments [38] while others showed double-stranded fragments [27].

With regard to cancer, Stroun et al., in 1987, isolated and characterized cirDNA found in cancer patients [46]. After dissociation of the original nucleoprotein complex, these authors showed that at that moment, the cirDNA was double-stranded and measured 0.5–21 kbp, revealing that cirDNA of cancer origin is smaller than genomic DNA. Since then, it is considered that cirDNA is made up of DNA fragments. Two years later, Stroun et al. identified specific properties of the cirDNA of cancer origin showing that the double-stranded DNA of tumor origin is less stable than that derived from non-tumour cells [22]. Advanced technologies, as well as data resulting from investigation of the release mechanisms, enabled further understanding of the cirDNA characteristics and origins.

#### A more precise structural identification: “signature” of the release mechanism

**Fig. 2:**
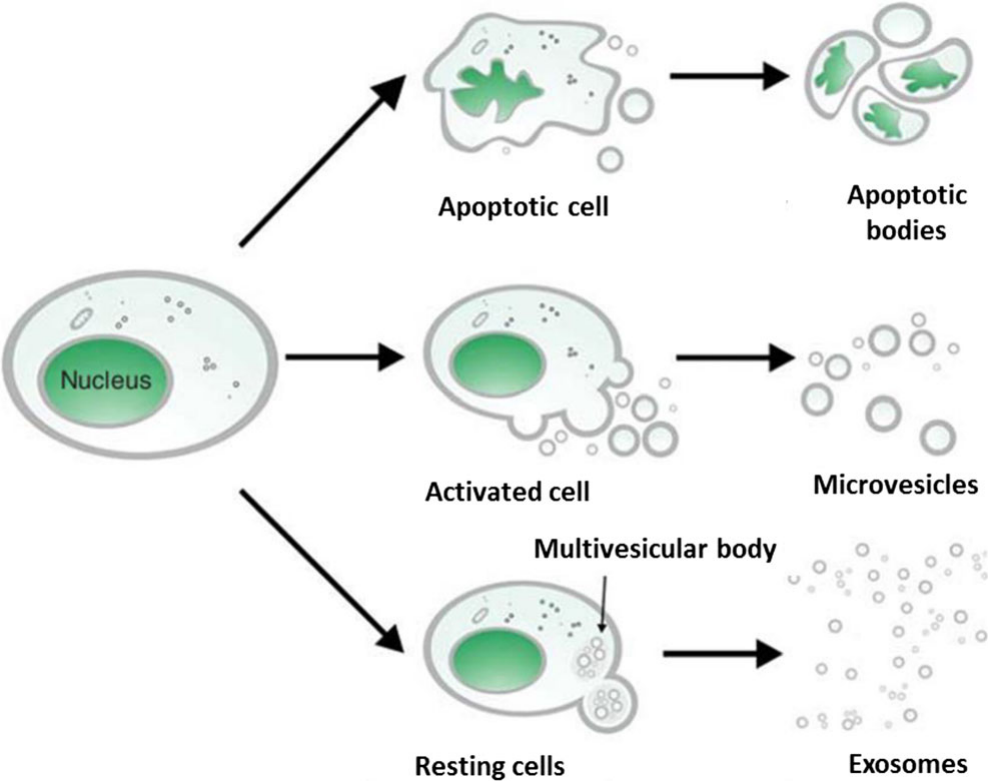
Potential vesicular structures of circulating DNA (from Rykova et al. [48])

DNA is a very electrostatic molecule with properties of auto-condensation and an ability to complex with other molecules or structures [47]. It has been shown that cirDNA is present either in molecular or macromolecular complexes or internalized in vesicles [48, 49] (Fig. 2). Such structures protect it from nucleases present in the circulatory system and reduce recognition as a danger signal by the immune system [50]. cirDNA can equally be attached to the exterior of the cell membrane from which it can be detached and so be freed in the circulatory system [48, 49]. Different mechanisms can be envisaged that permit the translocation of DNA from the intracellular to the extracellular compartment yet remaining biologically stable. The structural description of cirDNA is reflected in its origins: apoptosis, necrosis, phagocytosis, oncosis, and active secretion have all been evoked and linked to particular structures. The mechanism of active secretion was demonstrated by M. Stroun, P. Gahan, and P. Anker leading to the discovery of the virtosome [51, 52]. Their studies on non-dividing cells such as lymphocytes and frog heart auricles have shown a nucleoprotein complex that was synthesized and spontaneously secreted through a regulated mechanism [19, 53–62]. Abolhassani et al. have equally shown an active secretion of extracellular DNA from the HL60 cell line [63]. Moreover, it has been found that fragments of cirDNA found in cancer patients contained the hTERT and hTr sequences that are not found in the genetic material of apoptotic cells [61].

#### Cellular origins of tumor cirDNA

##### General considerations

The rate of solid tumour progression is a long process of some 10 years. Three parameters affect tumor growth kinetics: these concern tumor doubling time, the tumor proliferation coefficient, i.e., the number of proliferating cells, and lastly, the level of tumor cell loss factor [64]. Concerning colorectal cancer, theoretical data indicate that the tumor doubling time is 90 days, the cell loss factor is of 96 %, and the level of cell proliferation is 15 % [65]. The doubling time and the level of cell loss are particularly high, explaining both the long time for the appearance of this cancer and its Gompertzian growth [66]. The cell loss factor of 96 % is particularly interesting for our work: these cell losses are due to the phenomenon of cell death and indicates that this tumor could be at the origin of a massive liberation of DNA into the extracellular compartment. Bettegowda et al. reported a high variation of the cirDNA amount in blood of patients depending upon the types of cancer with bladder, colorectal, ovarian, pancreas, and breast cancer exhibiting more than fivefold and tenfold amounts as compared to thyroid and glioma cancer, respectively [67]. Over and above the mechanisms at the origin of DNA release into the bloodstream, it is necessary to consider the cellular origins of the DNA found in the blood of cancer patients. The identification of genetic alterations in cirDNA specific to cancer cells has permitted the demonstration that the tumor cell compartment was a source of cirDNA. Nevertheless, the basal amount of cirDNA found in healthy individuals indicates that other non-tumour cells constitute a source of DNA release. In addition, a tumor is composed of malignant tumor cells, but equally of an ensemble of cells constituting the tumor microenvironment. Thus, stromal cells, endothelial cells, lymphocytes, and other immune cells equally constitute a potential source of cirDNA release in relation to tumor progression [47].

Three cellular sources of cirDNA can be found in a cancer patient:

- “Healthy” cells
- Malignant cells
- Tumor microenvironmental cells

**Fig. 3:**
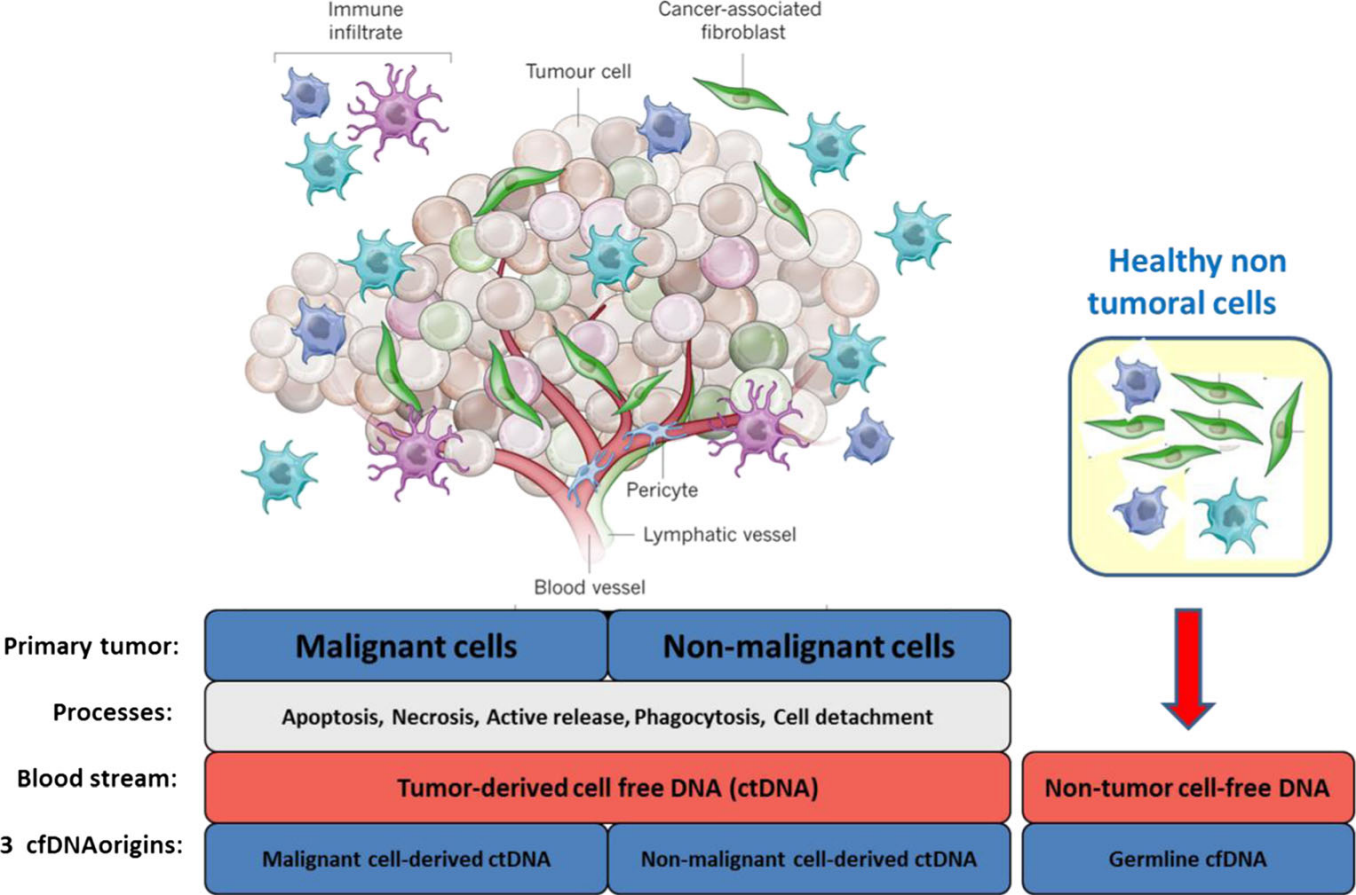
Distinct cellular origins of circulating DNA found in the blood of cancer patients

Each of these compartments may be subjected to different processes generating the release of different forms of DNA into the circulatory system. Figure 3 gives a resume of the cellular sources and mechanisms of release of cirDNA found in cancer patients.

##### Tumor cell origin of cirDNA

**Fig. 4:**
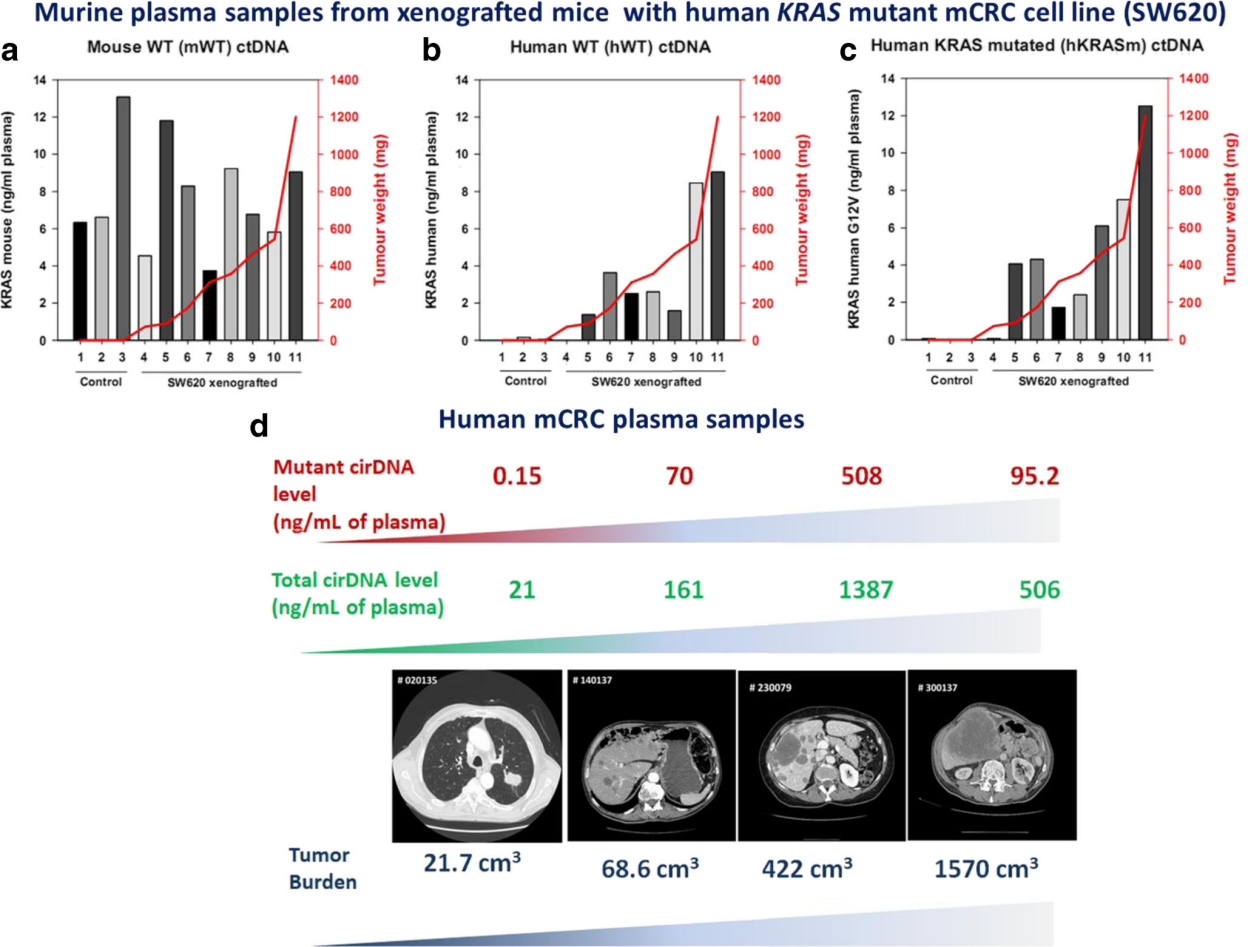
Circulating DNA levels correlate with tumor burden. **a** SW620 xenografted mouse model. Quantification by Q-PCR of cirDNA derived from malignant and non-malignant cells in the mouse model. Tumor weight is represented by the *red curve* (right axis). Concentration of cirDNA derived from mouse (normal) cells (mWT cirDNA) in control (not grafted) mice (mouse nos. 1–3) and in athymic nude mice (mouse nos. 4–11) xenografted with the SW620 colorectal human cells, determined using a primer set targeting a mouse *KRAS* second intron WT sequence. **b** SW620 xenografted mouse model. Concentration of cirDNA derived from human cells (hWT cirDNA) using a primer set targeting a human *KRAS* second intron WT sequence. **c** SW620 xenografted mouse model. Concentration of cirDNA derived from human cells (hKRASm cirDNA) using a primer set targeting a human *KRAS* second exon sequence that contains the G12V point mutation present in SW620. **d** Clinical mCRC plasma samples. Correlation between total cirDNA level and mutant cirDNA level in 4 *KRAS* mutant mCRC patients (adapted from Mouliere et al [68] and El Messaoudi  et al. [74]) (color figure online)

**Fig. 5:**
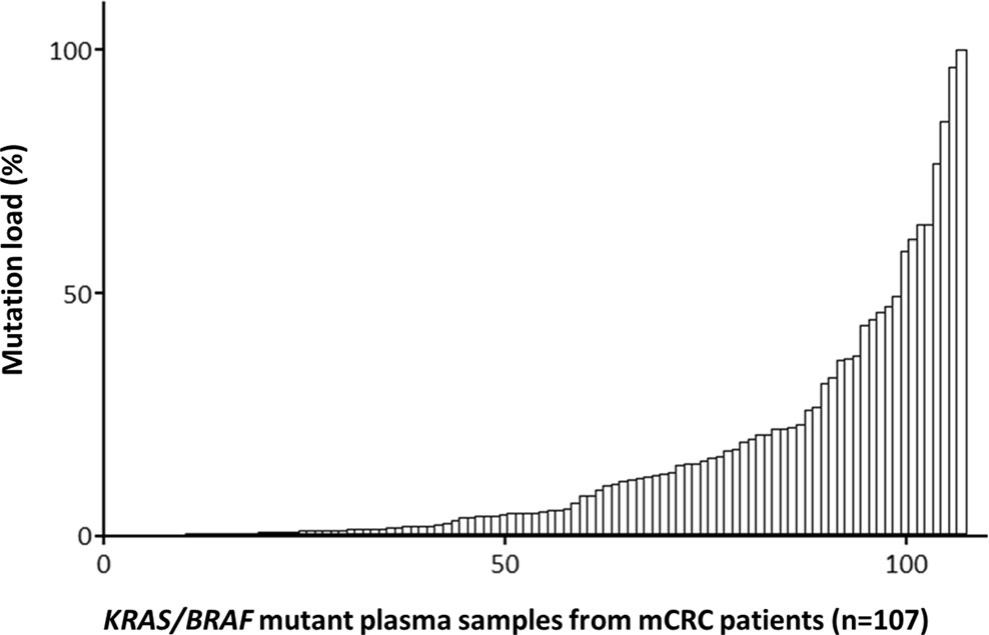
Strong interindividual heterogeneity of cirDNA mutation load values (mutant allele frequencies) (adapted from Mouliere et al. [68])

It has been demonstrated that the amount of cirDNA in the circulation compartment increases with tumor cell number increase. More elevated amounts are found in blood from advanced and metastatic cancer patients [67–71] than in early stage cancer patients. Higher levels of cirDNA (5–1500 ng/mL) found in cancer patients as compared to healthy individuals (1–5 ng/mL) [69, 72] result from the release of cirDNA both by malignant and non-malignant cells since the germline cirDNA level deriving from normal cells stays constant [47, 68, 73]. Therefore, elevated cirDNA levels may account for the tumor burden [68, 73, 74] (Fig. 4). It is reasonable to postulate that the total mutant cirDNA concentration accounts for the cirDNA deriving from malignant cells [74]. Thus, examination of the mutational load or the proportion of the mutant cirDNA within the total amount of cirDNA, first observed by Mouliere et al. [68–70, 74] and subsequently in various studies [67, 75], demonstrated that their respective amount varies greatly (from 0.003 to 95 %), highlighting a strong interindividual heterogeneity (Fig. 5). Moreover, in our studies, one third of the mutant plasma samples may exhibit a mutation load higher than 25 % [69, 70].

Hence, we suggest that either tumor cells variably release DNA as compared with the tumor surrounding stroma cells and normal cells or that mutant DNA analysis may depend upon tumor clonality. The detection of point mutation by quantifying the proportion of mutant circulating tumor DNA as presented here provides a powerful means toward assessing the proportion of cirDNA from different origins [47].

In spite of the numerous studies examining only circulating tumor-derived DNA, the total cirDNA concentration in the circulation compartment should be assessed since it better corresponds to the tumor mass especially when the total cirDNA concentration is high (i.e., > 20 ng/mL). It should be associated with the circulating tumor DNA level to better understand the tumor dynamics and the clonal heterogeneity over time and seems a valuable biomarker for tumor burden or tumor progression.

**Fig. 6:**
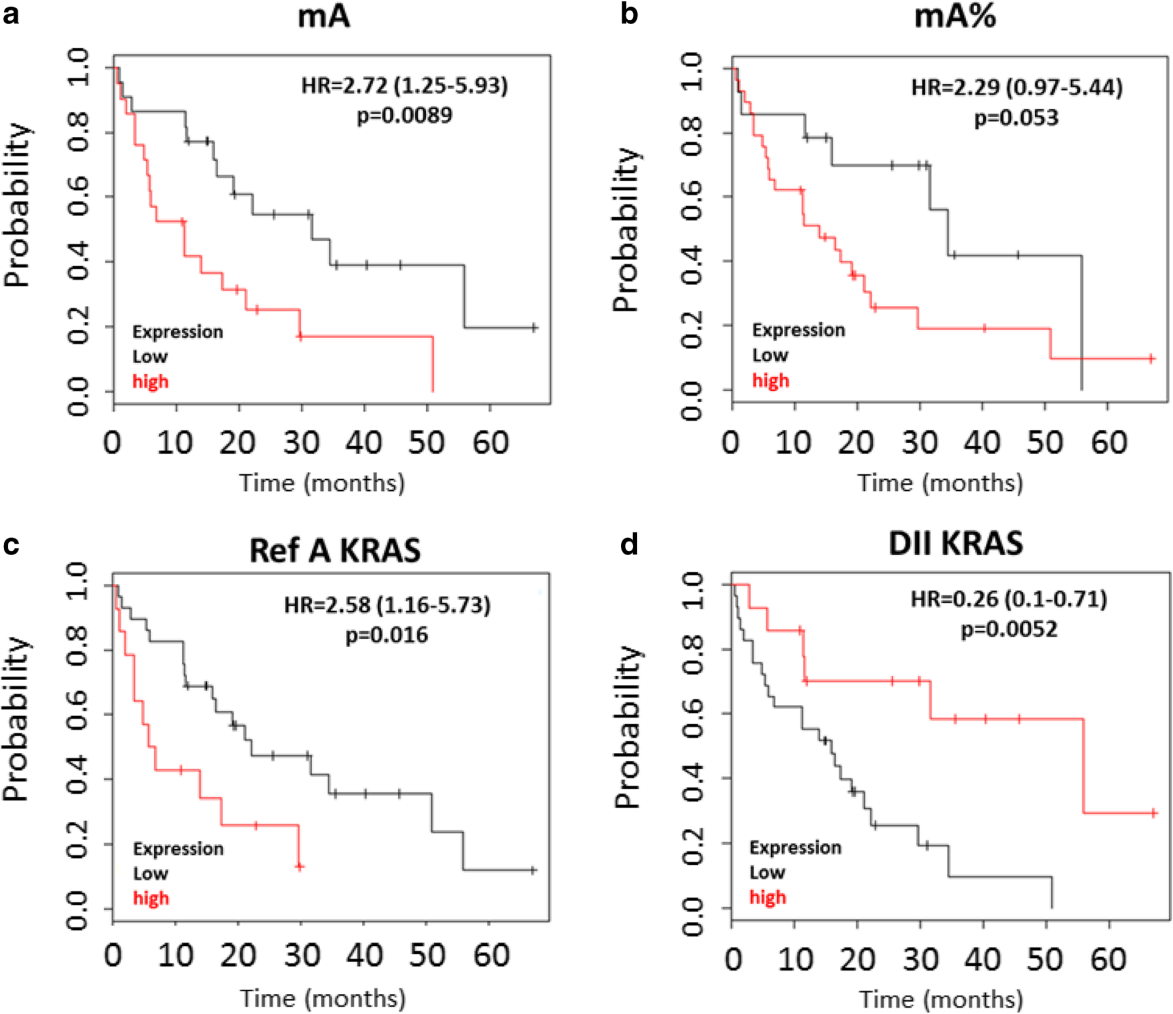
Correlation of circulating DNA parameters and overall survival. Overall survival analysis on a set of mCRC patients with *KRAS* or *BRAF* mutation. **a** Kaplan-Meier survival curve and log-rank test according to mA (mutant cirDNA concentration) determined by cirDNA analysis dichotomized around the median (3.06 ng/mL, *n* = 43). **b** Kaplan-Meier survival curve and log-rank test according to mA% (mutation load) dichotomized to the first tertile (4.14 %) determined by cirDNA analysis (*n* = 43). **c** Kaplan-Meier survival curve and log-rank test according to Ref A KRAS (total cirDNA concentration) dichotomized around the second tertile (107.0 ng/mL, *n* = 43). **d** Kaplan-Meier survival curve and log-rank test according to DNA integrity index (DII) determined by cirDNA analysis dichotomized around the second tertile (0.20, *n* = 43) (from El Messaoudi et al [74])

This is of particular importance when considering that a low proportion of malignant cells in colorectal cancer is related to poor cancer-specific survival [76]. In 2012, Spindler et al. showed the prognostic value of the total cirDNA concentration and mutant cirDNA concentration for a cohort of metastatic colorectal cancer patients [77]. Furthermore, our studies on metastatic colorectal cancer showed that high levels of total cirDNA and mutant cirDNA were strongly correlated with diminution of overall survival as well as high levels of mutation load (mutant allele frequency) and high cirDNA fragmentation level [74] (Fig. 6). An investigation toward deciphering the role of the tumor environment cells in other endothelial cancers or other cancer types and in primary versus metastatic tumor tissue would benefit from distinguishing between the concentration of mutant and non-mutant cirDNA. This former observation might account for the role of autophagy and hypoxia on cirDNA release either in respect to their structural forms or amount released in the blood stream [78]. Both physiological conditions are linked to the tumor microenvironment. Autophagy is associated with citrullination of histones to allow for the unwinding and subsequent expulsion of DNA. Autophagy also ensures the physiological turnover of old and damaged organelles being an adaptive response under stressful conditions. As such, autophagic activity regulates apoptosis and, as a consequence, cirDNA release. Hypoxia is proposed as a major process involved in tumor growth, invasion, and metastasis [79]. Malignant cells could activate autophagy as a survival mechanism, allowing an alternative energy source from their self-digestion. Conversely, Sato et al. revealed that while autophagy was activated in malignant cells, it was not activated in cells from the microenvironment [80]. This phenomenon could explain the high level of specific malignant-derived cirDNA observed in some cases. CirDNA release was found to be clearly dependent upon hypoxic conditions [79] and could be of diagnostic interest for solid tumors especially for tumors with an extreme hypoxic signature such as pancreatic cancer, and so could be proposed as a marker for the screening or surveillance for pancreatic cancer [81].

##### Mitochondrial-derived cirDNA

In addition, mitochondrial-derived cirDNA (cirmtDNA) has been found in the blood of healthy subjects and patients with various diseases, especially in cancer patients [82]. Important levels of cirmtDNA copies have been found in the blood of both cancer patients [83, 84] and healthy individuals [85]. Mitochondrial DNA is released in the circulation following general mechanisms of cell death and specific mitophagy cell death [86]. Their circulating structures are poorly known: however, it seems that they are present as either bound to internal and external mitochondrial membrane fragments or as intact cell-free mitochondrial DNA [87]. Nevertheless, analysis of cirmtDNA appears now to be of great importance with numerous studies showing diagnostic applications in various diseases (e.g., diabetes, acute myocardial infarction, atherogenesis, granulomatosis with polyangitiis) [88–91], physiopathological conditions (e.g., trauma) [92], or physiological states such as intense effort [93, 94]. Only a few clear crucial observations are made in the field of oncology with regard to theragnostics, prognosis, or patient follow-up [82, 83, 85, 95–97], but several publications converge to state the need for assaying cirmtDNA in order to associate its level either with inflammation processes [92, 98, 99] or the extracellular DNA TRAPs phenomena [100] which recently appeared to have an identified role with regard to tumorogenesis and metastasis [101, 102] (see paragraph toward Sect. 3.4). Due to the multiple copy characteristic of this DNA (many dozens of thousands of copies per cell), it seems judicious to be interested in cirmtDNA detection that appears to be an important source of cirDNA. In addition, important mitochondrial DNA somatic mutation level and important level of copy number variation have been reported for a number of cancers [103–105] and could be analyzed via cirmtDNA. In this context, we have initiated studies on cirDNA of mitochondrial origin showing that cirmtDNA is present in major amount in the blood (unpublished data). The determination of the mean values of cirmtDNA is currently under study with samples from different cohorts, permitting a tight statistical analysis. We have also performed experiments using samples from xenografted mice revealing that tumor and non-tumor cirmtDNA concentrations are elevated (unpublished data). Interestingly, we have shown that human tumor cirmtDNA is more fragmented than mouse non-tumor cirmtDNA (unpublished data). The experiments employing clinical samples show a level of integrity close to one in both cancer patients and healthy subjects (unpublished data): This highlights the important structural differences with nuclear cirDNA. It is already possible to imagine this difference by the fact that the mitochondrial DNA is a small DNA (16,000 bp), circular and unprotected by histones, that which excludes the possibility of a nucleosomal structure unlike the cirDNA of nuclear origin. Very few studies have considered the fragmentation of cirmtDNA: the experiments of filtration and successive centrifugations using the plasma from healthy individuals seem to indicate that cirmtDNA is composed of associated and non-associated particles elements [87]. Ellinger et al. showed the integrity to be between 0.5 and 1.0 for healthy individuals and urological cancer patients [83]. Another study showed the integrity index to be close to one for healthy individuals and more elevated than in carcinogenic agent exposed patients [85]. Same observation was made between healthy individuals and clear cell renal carcinoma patients [106]. A work of Jiang et al. showed that cirmtDNA was shorter than nuclear cirDNA in cancer, cirrhotic, HBV-infected patients, and healthy subjects [107]. This work did not show cirmtDNA structural differences between the healthy group and the affected patients [107]. These data imply the presence of a “stable” structure, raising questions concerning the identification of these structures: are they the signature of the presence of mitochondria in the blood? Are they especially stable lipoprotein complexes? A positive correlation between the number of platelets and the cirmtDNA concentration has also been documented [87]. Nevertheless, the reported data are actually insufficient and it is necessary to make a systematic study of the structure of this DNA employing well-established mouse models and large cohorts of clinical samples.

##### Specific cellular cirDNA origins

Unsuccessful attempts were made in the past to decipher the cirDNA origin by determining the sequence of cirDNA fragments [108]. However, Shendure et al. were able to determine the cell type of origin by generating maps of genome-wide *in vivo* nucleosome occupancy by using deep sequencing of plasma cell-free DNA [109]. Nucleosome spacing inferred from cirDNA in healthy individuals correlates most strongly with epigenetic features of lymphoid and myeloid cells, consistent with hematopoietic cell death as the normal source of cirDNA. Although this cell origin is still dominant among the cirDNA fragment population, this work showed as well malignant cell origin of cancer patient-derived cirDNA. A recent work of Lehmann-Werman et al. [110] involved a study of the specific tissue origins of cirDNA in various affected diseased patients using cirDNA methylation pattern analysis. They determined that cirDNA was derived from pancreatic β cells in insulin-dependent diabetic patients, that cirDNA came from oligodendrocytes in relapsing multiple sclerosis patients, and that after either cerebrovascular accident or heat attack, methylation analysis of cirDNA from those patients revealed a neuronal/glial origin. Finally, in either pancreatitis or pancreas cancer patients, they showed that it came from the exocrine pancreatic cells [110].

##### cirDNA is not derived from CTCs

Note, cirDNA does not derive from circulating tumor cells (CTCs) that constitute another biological source of the liquid biopsy approach. This postulate resides on the fact that there is a discrepancy between the number of CTCs and the quantity of cirDNA in the blood. As stated by Crowley et al. [33], “a single diploid human cell contains 6 pg of DNA and there is a median of 17 ng of DNA/mL of plasma in advanced-stage cancers; therefore, if CTCs were the primary source of ctDNA it would require over 2000 cells/mL of plasma. In reality, there are, on average, less than 10 CTCs per 7.5 mL of blood.” Our group and others [67] have confirmed this observation. Consequently, there are between 100 and 1000 times more Genome equivalents in cirDNA as compared to CTCs.

### Structures relevant to tumor-related cirDNA

#### The nucleosome

Apoptotic DNA cleavage produces a characteristic ladder pattern of 180–200 bp or multiples thereof (oligonucleosomes) of DNA fragmentation [108]. Apoptosis often leads to the degradation of chromosomal DNA. DNA fragmentation results from a caspase-activated DNase in the dying cells and by lysosomal DNase II after the dying cells are phagocytosed [111]. A nucleosome is composed of a histone octamer and double-stranded DNA turned about this protein complex that is stabilized by histone H1. Each nucleosome is linked to another by double-stranded DNA, the linker DNA. The DNA rolled around the histone octamer is 147 bp and the linker DNA is 20–90 bp. The association of these fragments assures a nucleosomal structural integrity that protects the DNA from enzymatic degradation in the circulatory system [112, 113]. The binding of the DNA around the protein complex is made by electrostatic interaction, the DNA being negatively charged and the protein positively charged. This fragmentation pattern could equally be the sign of a cell death mechanism due to oncosis involving a cell death mechanism known as ischemia [114]. It is characterized by mitochondrial and nuclear swelling followed by cytoplasmic vacuolization before cellular breakdown. Since 2001, Holdenrieder et al., using immunoenzymatic methods specific for nucleosomes, have confirmed their presence in the circulatory system of cancer patients with a lesser amount in healthy individuals [112, 113, 115].

The characterization of a size comprising between 160 and 180 bp of cirDNA remained a postulate for a long time. Nevertheless, more recent observations have shown that cirDNA of tumor origin is more fragmented than cirDNA deriving from healthy cells and with a size mainly smaller than 145 bp [72, 73, 109].

#### Microvesicles of tumoral origin

*In vivo* microvesicles are particulates made of one or several surrounding lipidic membranes encapsulating an aqueous compartment that may contain cellular constituents or molecules. An abnormally high number of microvesicles is secreted and found in the blood of cancer patients [47, 116] with many types of microvesicles being secreted by tumor cells (Fig. 2): exosomes, apoptotic bodies, and other more heterogeneous microvesicles. All of these structures contain tumor cell DNA. These microvesicles can possess a transforming capacity [117, 118] and constitute a key structure of cirDNA.

##### Exosomes

Exosomes are microvesicles measuring 30–100 nm and secreted by most cells [119]. These microvesicles have functional and biological properties, notably, the capacity for lateral transfer of material. The exosomes are composed of proteins and particular lipids as well as mRNA and microRNA [120, 121]. Recently, it has been demonstrated that exosomes also contain small amounts of DNA and exosomes tumor-derived DNA has been characterized [122]: this seems to exist in two main forms: one double-stranded form external to the microvesicle is bound to the membrane and is of a large size (>2.5 kbp). The other part of the double-stranded DNA, less important, is in the interior of the vesicle and measures 100 bp to 2.5 kbp. Subsequent extraction of miRNA or mRNA from exosome preparations from blood is considered as a means of obtaining a high level of those circulating nucleic acids toward developing a diagnostic panel of mRNAs/microRNAs. However, it is not clear, as to whether or not, there are more miRNAs in exosomes than in blood [121] and that the methods used for their extraction eliminate contamination by larger microvesicles.

##### Microparticles or ectosomes

The microparticles (MPs), or ectosomes, are membrane fragments measuring 200–1000 nm in diameter [120]. They are released into extracellular compartments by most eukaryotic cells. Their formation is the result of highly remodeled membranes accompanying the process of the activation of/or cellular apoptosis leading to an asymmetrical repartition of membrane phospholipids with externalization of phosphatidylserine to the surface of the MPs. They contain both DNA and RNA [123].

##### Apoptotic bodies

Apoptosis is described as a cell death mechanism characterized by its morphological changes, including cell shrinkage, membrane blebbing, chromatin condensation, and nuclear fragmentation. It is associated with fundamental cell phenomena such as cell survival as well as those that control proliferation and differentiation. Defects in apoptotic pathways are now thought to contribute to a number of human diseases, ranging from neurodegenerative disorders to malignancy. Apoptosis and the genes that control it have a profound effect on the malignant phenotype [124].

Membrane blebbing leads to release from cells of apoptotic bodies which are vesicles measuring between 1 and 5 μm formed during the late phase of apoptosis and corresponding to parts of a dying cell. Apoptosis induces cell cytoskeleton breaks causing the membrane to bulge outward. These protuberances may separate from the cell, encapsulating part of the cytoplasm, to become apoptotic bodies. These are then engulfed by phagocytic cells and their components recycled. They contain degraded DNA during apoptosis. The nucleic acids contained in these apoptotic bodies will be protected against the action of DNAses and RNAses [125].

#### Macromolecular structures

##### Virtosome or nucleic acid-lipoprotein complexes

The virtosome is a nucleic acid-lipoprotein entity discovered and identified by P. Anker, M. Stroun, and P. Gahan. Their first investigations revealed that non-dividing cells such as lymphocytes released DNA in their culture medium, confirming earlier lymphocyte studies [52, 60, 126–129]. Subsequently, Stroun and Anker showed that this released DNA was newly synthesized through ^3^H-thymidine labeling studies on lymphocytes and frog heart auricle pairs [60, 130] and that it was associated with RNA [131]. Since both nucleic acids were resistant to nuclease activity, it was considered that they were protected by lipoprotein. Protein presence was determined by RNAse activity affecting RNA only after treatment with proteinase k [131–133] while the presence of lipids was deduced from the complex’s behavior during density gradient centrifugation [133], the effects of freezing and thawing on the complex [132, 133], and the incorporation of radioactive phospholipid precursors [132, 134]. Later studies employing radioactive precursors demonstrated that the RNA, protein, and associated phospholipids were also both newly synthesized and synthesized at approximately the same time [131–133, 135]. This DNA/RNA-lipoprotein complex has been termed a virtosome and was suggested to be an intercellular messenger [51]. *In vitro* studies show the complex to be released in an apparently energy-dependent step [132], only from living cells [20, 132–134] and in a controlled manner [60, 130]. Labeling experiments have shown that the components appear in the cytoplasm at about 3 h after commencing labeling and that the complex is released from cells 3–6 h later dependent upon the origin of the cells studied, i.e., human, other mammalian, avian, amphibian, and plant cells [52, 60, 126–130, 132–134, 136, 137]. The above studies (except for those in references [132–135, 137]) were performed on the supernatants from the culture media that had been centrifuged, initially, to remove nuclei and cell debris followed by mitochondria and lysosomes prior to ultracentrifugation at speeds varying from 120,000 to 340,000*g* to yield the supernatant. Purification was difficult since the complex did not pellet at these speeds, did not pellet on centrifugation in a cesium chloride gradient, and only partially migrated upward on a sucrose gradient [132]. Thus, further purification was achieved by agarose gel chromatography [133]. Studies of this fraction confirmed the previous findings of the presence of newly synthesized DNA, RNA protein, and phospholipids that separated in the same fractions from the agarose gel column [133, 135] confirming also the presence of a complex.

The complex isolated in this way from chick embryo fibroblasts also permitted the demonstration that the uptake of the complex into fresh cells did not modify the isolated fraction and that the complex remained the same both when released from cells and when taken up by fresh cells [133]. This implied that the complex did not have a standard limiting membrane. This result was confirmed by a similar study on the uptake by non-stimulated lymphocytes of the complex released from mouse tumor J774 cells [137].

##### Neutrophil extracellular DNA traps

**Fig. 7:**
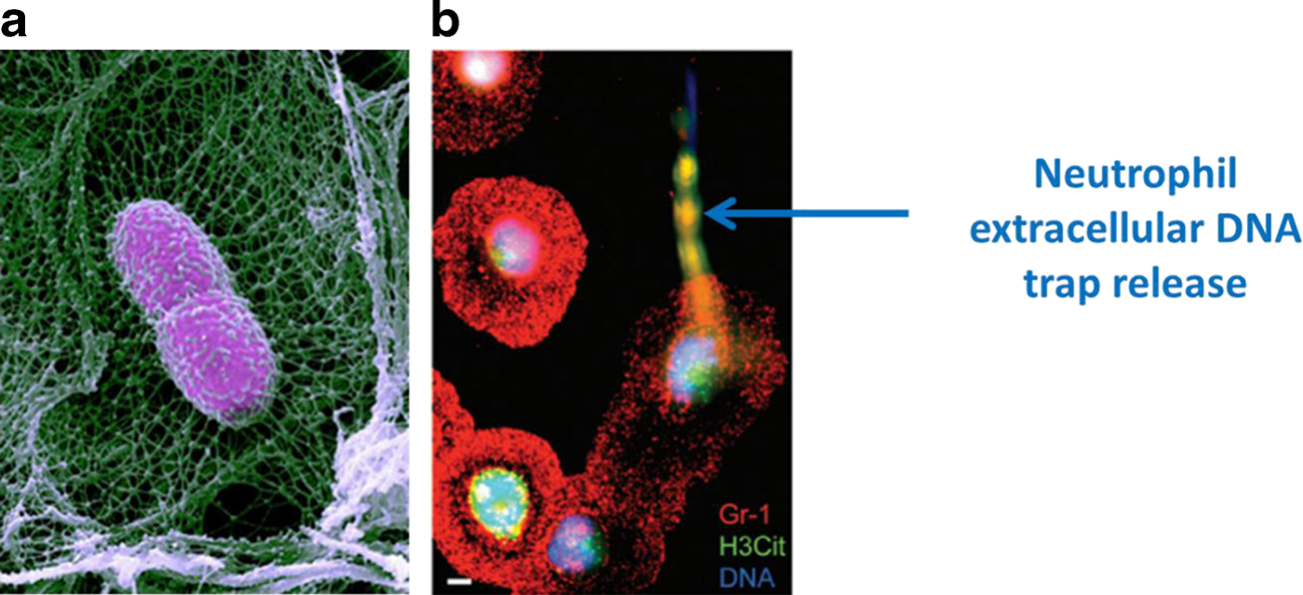
Neutrophil extracellular DNA traps. **a** *Klebsiella pneumonia* bacterium (*pink*) snared in a neutrophil extracellular trap (*green*) in a mouse lung. Credit: Papayannopoulos et al. (image by Volker Brinkman and Abdul Hakkim). **b** Immunostaining double-labeling of neutrophils releasing NETs isolated from tumor-bearing mice following 1 h activation with calcium ionophore (*scale bar* = 5 μm) (from Demers and Wagner 224)

Neutrophils represent a significant portion of the tumor-associated inflammatory cell infiltrate [138]. It has been shown that the tumor-associated neutrophils (TANs) demonstrate pro-tumorigenic as well as anti-tumorogenic properties related to their activation stage [139–141]. NETosis by cell lysis is a cell death program of neutrophils which occurs when neutrophils are activated by pathogen agents or particular conditions: NETosis leads to chromatin decondensation, lysis of cell and nuclear membranes, and finally the release of neutrophil extracellular DNA traps (NETs) [142]. NETs are composed of remodeled extracellular DNA fibers and anti-microbial granules initially present in the neutrophil [143]. This particular structure is dedicated to both trap and kill pathogens (Fig. 7) [144]. It has been shown that NETosis by cell lysis can be opposed to the “vital NETosis”, leading to release of DNA without the cell lysing [145]. This may be activated under specific conditions. The NETs, identified in 2004 [142], thus constitute, in part, one of the origins of that which is called cirDNA. Liberated under conditions of either infections or particular pathophysiological states, these structures are equally interesting from the functional point of view since it has been shown that they possess biological and particular pathophysiological effects notably in autoimmune pathologies, cancer, sepsis, thrombotic illnesses, and in the inflammatory response [101, 146–150]. These situations are especially ones in which high levels of cirDNA have been reported. It has also been shown that after intense physical exercise, the neutrophils secrete high levels of NETs, a situation in which high levels of cirDNA have also been reported [151]. In this context, one cannot completely exclude that myocyte death is equally at the origin of the release of cirDNA.

##### Eosinophil extracellular DNA traps

As eosinophils are also present in the tumor-associated inflammatory cell infiltrate [152], it seems interesting to consider eosinophil extracellular DNA traps (EETs) released from eosinophils under activation [153]. Eosinophils stay viable after the release of EETs indicating particular mechanisms of release. They contain only mitochondrial DNA secreted in a catapult-like manner as opposed to neutrophil NETs that contain both nuclear and mitochondrial DNAs, indicating that this secretion is really an active event and not a release following cell death [153, 154]. Inflammation has long been associated with the development of cancer, in particular, with regard to the maintenance of tissue homeostasis and repair [155]. So far, EETs were not shown to be associated with cancer.

##### Circulating DNA linked with serum protein

DNA, being a very electrostatic molecule, is able to link with proteins present in the circulatory system to form complexes [47, 48, 156, 157]. This was described to occur with some abundant proteins such as either albumin or immunoglobulins and less abundant serum proteins such as fibronectin and the C1q complement component. Particular pathological situations influencing the serum protein levels can cause a fluctuation in the amount of cirDNA.

##### Circulating DNA linked to the cell surface

Some DNA and RNA are found at the surface of both leucocytes and erythrocytes in the blood [48, 49, 158]. *In vitro*, DNA fragments of 20 kbp are equally found at the cell surface [159]. This large size may be explained by either a particular secretion/release mechanism or by the fact that nucleases can have limited access to this DNA. This DNA linked to the cell surface can be either naked or associated with vesicular structures or linked to macromolecules as previously described. At the cell surface, there are some receptors capable of binding DNA, but equally to allow it to enter cells [2]. It has been shown that the nucleosomal structure was capable of crossing the cell membrane [50, 125]. Some proteins, such as albumin, are able to complex with cirDNA and favor its internalization into the cell via endocytosis [125].

#### Size of cirDNA in cancer patients

##### Unclear distribution of cirDNA size in cancer patients

**Fig. 8:**
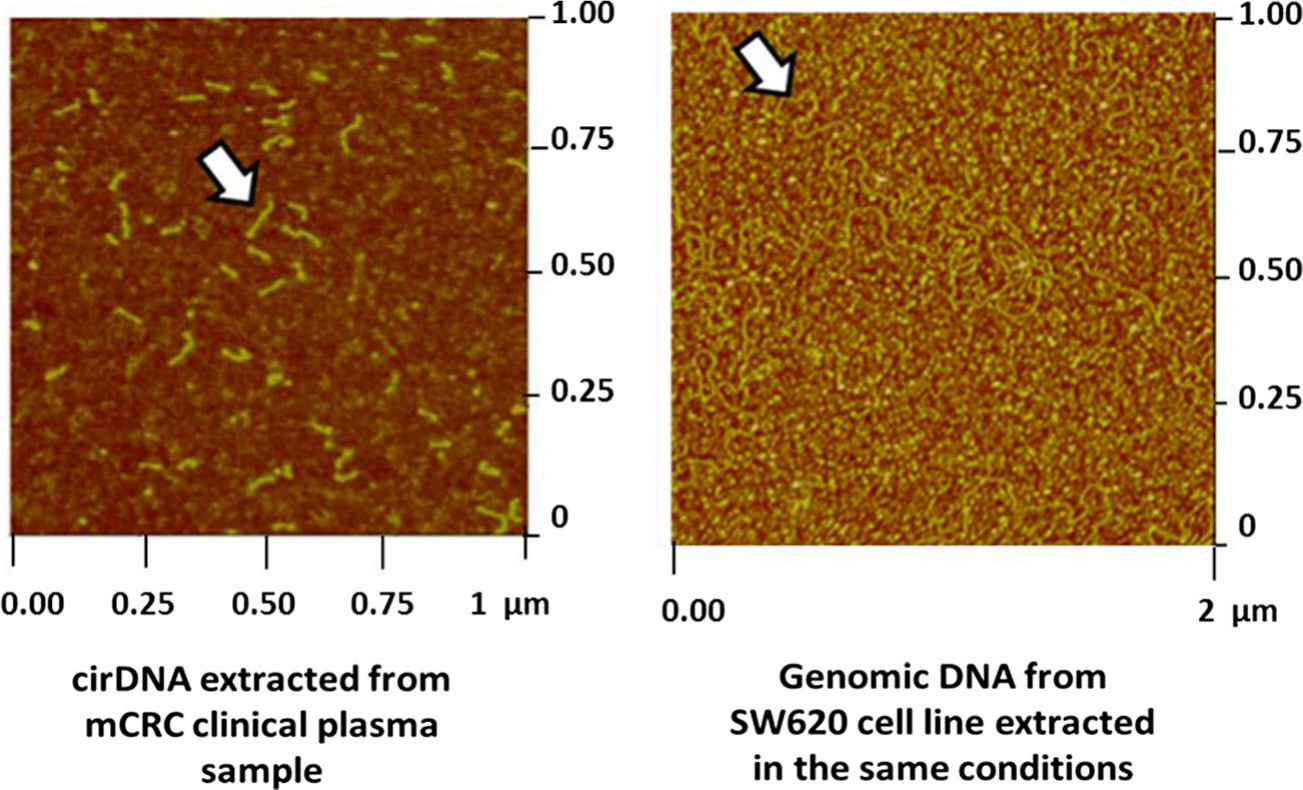
Vizualization of circulating DNA by atomic force microscopy. Circulating DNA extracted from a metastatic colorectal cancer patient plasma sample vizualized by atomic force microscopy (AFM) and compared to SW620 genomic DNA extracted in the same conditions than circulating DNA (adapted from Mouliere et al. 69)

Knowing cirDNA size is of great interest for a better understanding of their structure(s) and origin(s) that can be related to a particular tumor physiological/biological state, leading to a higher diagnostic value. Unfortunately, the lack of harmonization of the methods used for pre-analytical procedures has led to many controversies in the literature and has not permitted the drawing of clear conclusions in this area [1, 160–163]. As has already been discussed, the most reported size corresponding to mononucleosomes and oligonucleosomes [1, 108, 112] in the literature, has become the basic premise concerning the structure of cirDNA for many years. Nevertheless, other studies have described results that do not agree with this concept. Many studies have shown the presence of large-sized fragments of many kbp [48, 108, 125, 164, 165]. The presence of fragments of this size could be the signature of a mechanism of necrotic release. During necrosis, the chromatin chain is degraded in an anarchic way and can lead to fragments of >50 kbp. In contrast, we have shown by using either a Q-PCR-based method or AFM that cirDNA in cancer patients has a size mainly inferior to 145 bp [68, 69, 72, 166] (Fig. 8).

##### Circulating DNA of cancer patients is more fragmented than that of healthy individuals

**Fig. 9:**
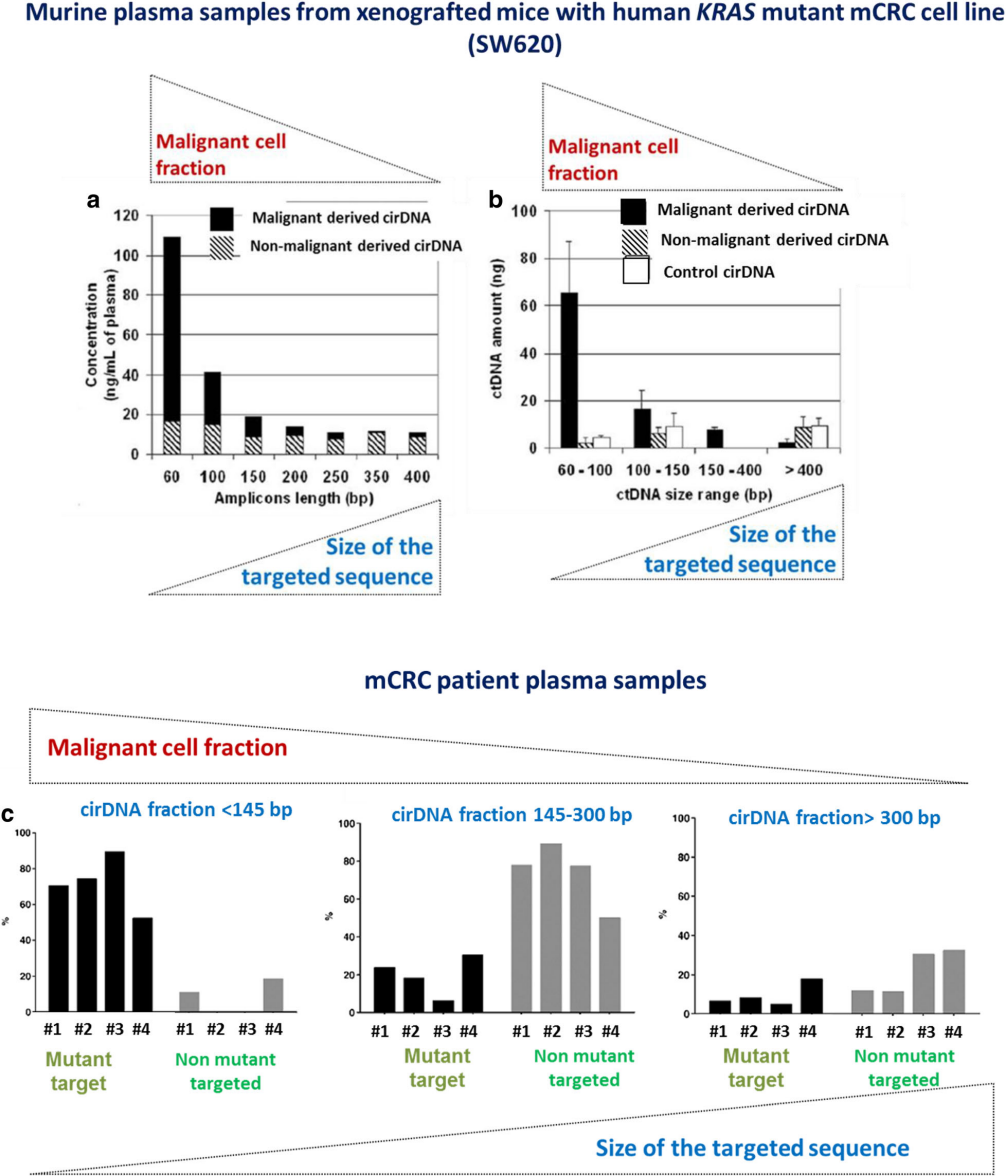
Malignant-derived circulating DNA is more fragmented than non-malignant derived circulating DNA. SW620 xenografted mouse model: **a** malignant (*black bars*) and non-malignant (*hatched bars*) cirDNA mean concentrations are expressed as ng/ml of plasma; the *bar height* is the sum of malignant and non-malignant cirDNA concentrations (estimated as the total ctDNA concentration). CirDNA was quantified by amplifying WT *KRAS* exon 2 sequences of increasing size: 60, 100, 150, 200, 250, 350, and 400 bp. **b** Fractional fragment size distribution of cirDNA amount from malignant and non-malignant cirDNA in xenografted mice and control cirDNA in non-xenografted mice. The cirDNA amount was arbitrarily estimated for the 60–100-, 100–150-, 150–400-, and >400-bp fragment size ranges. Clinical samples: **c** comparison of the cirDNA fragment size distribution of mutant (*black bars*) and WT (*gray bars*) cirDNA in four plasma samples from *KRAS* mutant mCRC patients. Mutant cirDNA was quantified by amplifying mutant *KRAS* exon 2 sequences of increasing size from 60 to 390 bp. Non-mutant cirDNA was quantified by amplifying WT *KRAS* exon 2 sequences of increasing size from 60 to 390 bp (adapted from Mouliere et al. [68, 72])

**Fig. 10:**
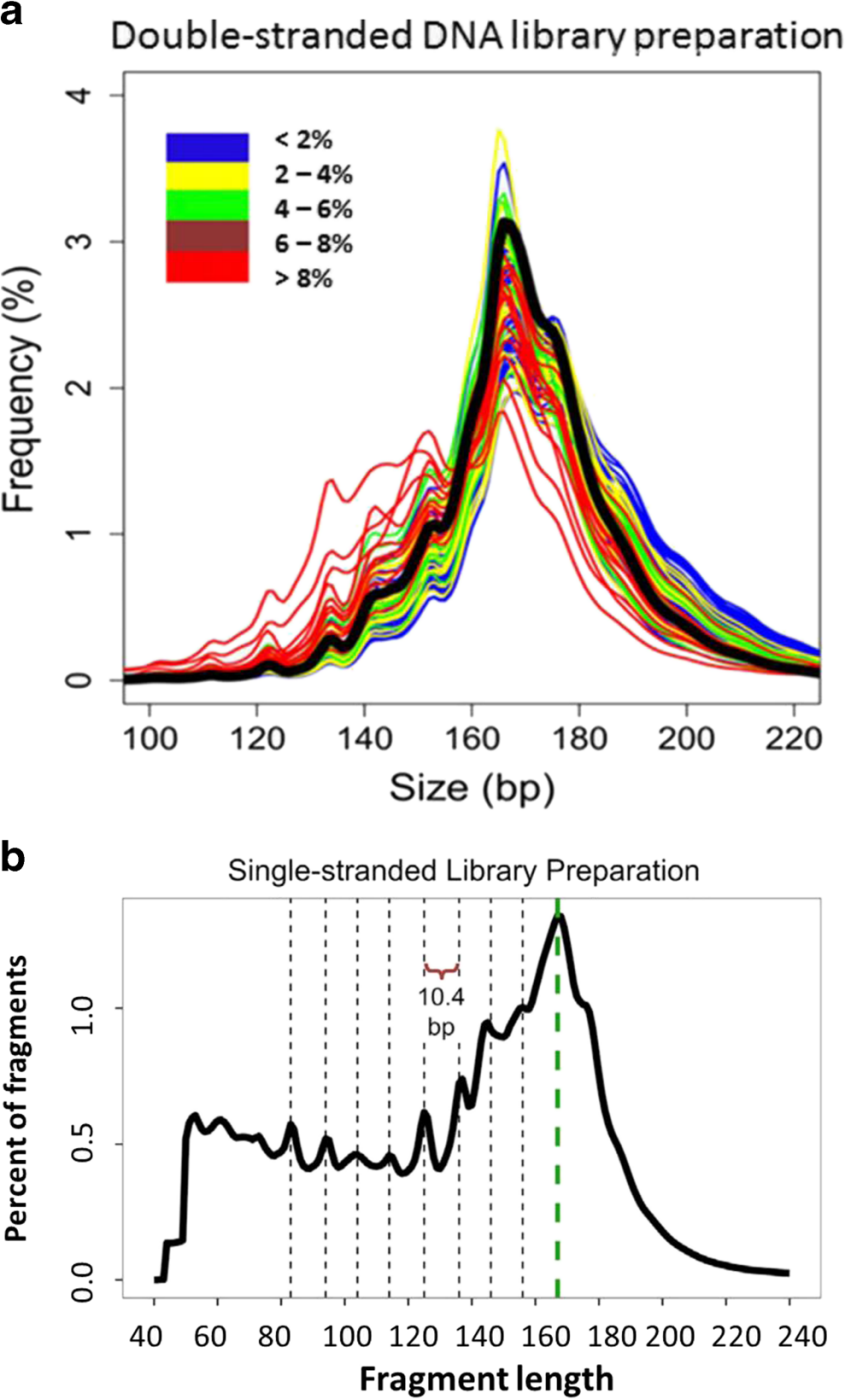
**a** Size distribution of malignant-derived circulating DNA. Ninety hepatocellular carcinoma patient plasma samples were examined by paired-end massively parallel sequencing. Different malignant-derived fractional concentrations were determined in HCC plasma samples (from <2 to >8 %). While size distribution peaks at 166 bp, it shifted progressively to the shorter size when malignant-derived cirDNA fraction increases (from Jiang et al. [107]). **b** Size distribution of circulating DNA in plasma sample from healthy individual as determined by next-generation sequencing from a single-stranded library preparation. Short fragments of 50–120 bp are considerably enriched as compared to conventional library preparation (from Snyder et al. [109])

Before developing a reliable tool for the analysis of cirDNA from cancer patients, our group has systematically analyzed the structure of tumor-derived cirDNA using an animal model and cancer patient samples, an approach not previously made [68, 73]. These studies have shown for the first time that tumor-derived cirDNA is highly fragmented and mainly composed of fragments <145 bp (Fig. 9). In parallel, the analysis of tumor cirDNA showed it to be more fragmented as compared to cirDNA from healthy individuals. These observations have disagreed with those previously described in the literature, indicating that the preponderant cirDNA size was 160–180 bp for cancer patients. However, several years later, our work was confirmed by an elegant study by Denis Lo’s group who, using a massively parallel sequencing-based method, showed the lower size mean of cirDNA from hepatocellular carcinoma cancer patients [107] (Fig. 10a). A recent work of Shendure et al. [109] remarkably studied fragmentation patterns of cirDNA and revealed that they constituted specific signatures of tissue origins of cirDNA. Moreover, their acute deep sequencing and library preparation methodology was designed to bypass the limits of a conventional DNA library in order to recover damaged and short double-stranded DNA fragments. They revealed that, even if there is a dominant structure of 167 bp, a high proportion of cirDNA fragments was below 167 bp [109] (Fig. 10b). This strong result highlights the need to elucidate the structures of cirDNA so as to improve their detection and analysis.

The observation that the sizes were mainly smaller than 145–160 bp calls into question apoptosis as the only origin of cirDNA. The description of this size range indicated that degradation, either enzymatically or by phagocytosis, occurred in the circulatory compartment after DNA liberation by either cell death or active secretion [47].

It seems that there are different cirDNA entities in the blood of healthy and cancer subjects and that their respective discrimination might be of valuable diagnostic value. In the light of the prenatal fetal chromosomal aneuploidy test in pregnant women, cirDNA size analysis could be used synergistically with a counting approach to improve the accuracy of the detection and cancer monitoring [68, 69, 107].

##### Size determination versus structure and origins

The many structures and mechanical origins evoked at the start of this review showed that cirDNA is a complex entity. Sizing following cirDNA extraction cannot fully account for characterizing their structures because it results from numerous biological and analytical variables. However, it is clear that knowledge on size and fragmentation are keys in accurately detecting and quantifying cirDNA, either total cirDNA or a fraction of cirDNA of various origins (mitochondrial, nuclear, tumor or healthy cells, tumor microenvironment cells, metastatic cells). In the light of this observation, works on cell-free DNA collected from other body constituents should rely on size specifics to their body origin rather than on blood DNA data.

### Subsequent fate of circulating DNA in the organism

The observations made on the structures and origins of cirDNA are not just the result of the mechanisms described earlier concerning the release of DNA into the circulatory system. They are also conditioned by the subsequent fate of the cirDNA in the organism, notably in the blood compartment.

Before considering the functional aspects of cirDNA, it is necessary to consider its subsequent fate in the organism after its release even though there are few publications on this subject. Such data have been determined mainly through either plasmid DNA or genomic DNA extracted from cell tissues. These DNAs were shown to be rapidly degraded by nucleases present in the blood and rapidly metabolized/eliminated by the liver and kidneys. Very little work has been focused on cirDNA sensu stricto in its native state: among eight women about to give birth, Lo et al. in 1999 determined that the mean half-life for the elimination of cirDNA of fetal origin was 16.3 min [167]. They have shown elimination to be in two phases: firstly, a slow elimination phase corresponding to a distribution/elimination phase followed by a second, faster phase corresponding to probably total elimination. In 2013, using massively parallel sequencing in post-delivery women, they confirmed a two-phase kinetics of fetal cirDNA. In this study, they identified a first phase with a 1 h half-life time and a second phase with a 13 h half-life time. They identified that even if trans-renal excretion was involved in the process of cirDNA elimination, it was not the major elimination mechanism [168]. Diehl et al. have determined on only a single colorectal cancer patient an elimination half-life time of 114 min after surgery [169]. These data showing discrepancies are currently insufficient and the determination of the subsequent fate of cirDNA in the organism is crucial for a better understanding of its role in the organism and its turnover so as to develop a tool for biomedical follow-up.

### Discussion on the structure and origin of cirDNA in respect to application in oncology

**Fig. 11:**
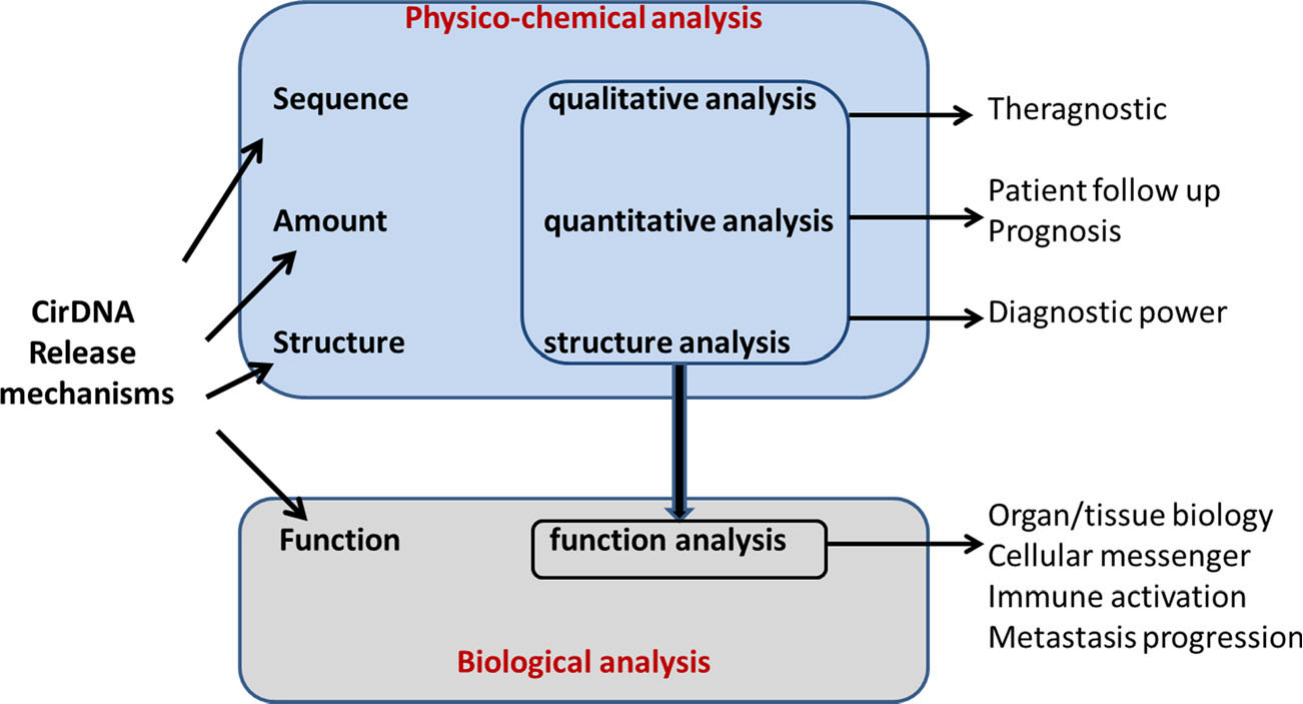
CirDNA structure, origin, and function as regard to its analysis toward clinical application. Physicochemical analysis of cirDNA, i.e., analysis of its sequence, its quantity, and its structure brings qualitative and quantitative information useful for theragnostics, diagnostic, prognosis, and follow-up in the context of cancer management care. Analysis of the functions of cirDNA acting as an intercellular messenger, an immune activator, and a mediator in metastatic progression provides useful information for the follow-up of cancer patients by cirDNA analysis

Due to the various cirDNA structures involved, and the subsequent various stability and fate, it is very difficult to determine with full accuracy the respective proportion of each structural entity. Such evidence would provide crucial information about tumor biology, for instance, to distinguish the evolution between the primary tumor and metastasis. In addition, examining cirDNA structures respective proportions may shed a new light on tumor dynamics, especially for following tumor progression, and so permit learning more about tumor aggressiveness in order to individually guide and tailor treatment for cancer patients (Fig. 11). On the other hand, specifically extracting cirDNA based upon specific vesicular compartments or molecular or intermolecular structures might be of great interest in terms of its diagnostic power in some pathologies or in some cancer phases such as metastasis (Fig. 11).

Hence, in addition to the work performed on detecting specific sequences resulting from oncogenetic phenomena such as either point mutations or aberrant methylation that are mostly directed toward clinical applications such as theragnostics, it appears that the quantitative determination of malignant cells derived cirDNA as well as of non malignant cells derived cirDNA is of great interest (Fig. 11). Scrutinizing the structural heterogeneity of cancer patient, cirDNA analysis could paradoxically, be a great opportunity to delineate diagnostic tools of higher performance.

## Functional aspects

Two different striking discoveries with regard to the cirDNA functional aspects were observed: the capacities as intercellular messenger and as genometastasis. This paragraph will also describe the immunological properties of ccfDNA.

### Capacity as intercellular messenger

#### Discovery and concept

**Fig. 12:**
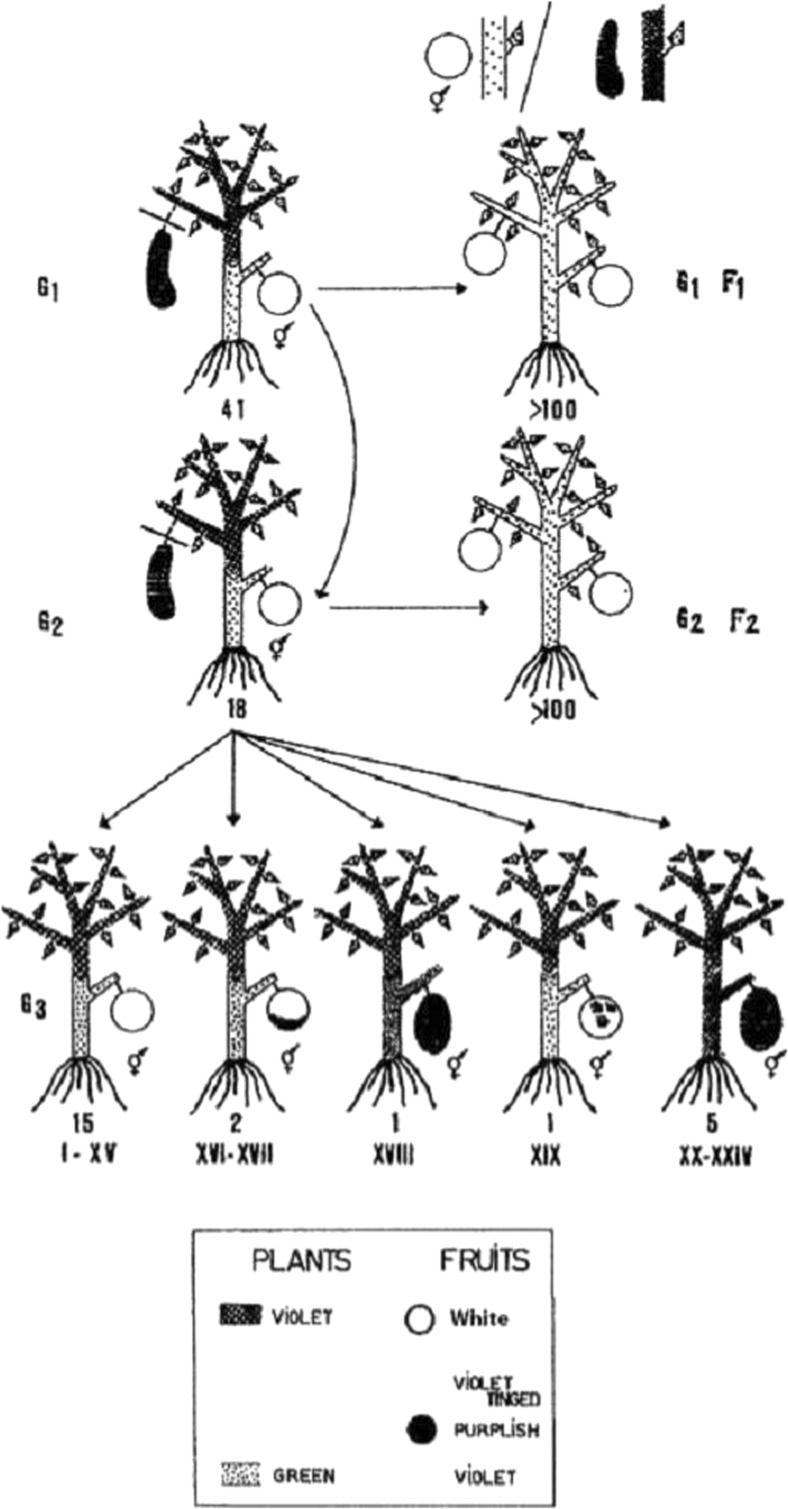
Demonstration of transmission of inherited characters by hybridization in eggplants. Heterografts between *S. melongena* variety Long violet (mentor plant) and the variety White round (pupil plant) (from Stroun [16])

The story began in the 1960s with the work of M. Stroun, P. Anker, and P. Gahan in the context of an ideological war over the Darwinian theory between the Russians and the West [170–172]. Russian researchers were interested by a commentary of Darwin concerning the nonsexual transmission of characters through intervarietal vegetal graft experiments performed by a number of English researchers [173, 174]. This concept was also supported by experiments with blood transfusion indicating the presence of a circulating entity carrying genetic information and with a transforming capacity [175]. This notion recalled that of Darwin’s “gemmules” structures circulating throughout the organism to reach the germinal cells where they would permit the transfer of genetic information either directly to the F1 generation or to the F2 generation with the omission of expression in the F1 generation. This allowed Darwin to explain how inherited features could “jump” a generation. The gemmules were defined as self-replicating structures that could also give rise directly to cells [173]. M. Stroun repeated the Russian grafting experiments using eggplants in the first approach and confirmed the transmission of acquired characters (Fig. 12). He proposed that the nucleic acids are active carriers of genetic information that circulate between the graft and the reproductive cells of the stock that are responsible for the transmission of genetic characters [16]. He also repeated blood transfusion experiments from Guinea hens to white leghorn chicken and observed alterations of the feather color in white leghorn chickens [176]. From that time, the idea of cirDNA was born [16, 177]. M. Stroun and P. Anker repeated these experiments on different plants and higher organisms and obtained positive results. They demonstrated notably that on dipping the cut shoots of a tomato plants in bacterial DNA, this foreign DNA was found throughout the shoots attached to the host DNA [59]. Subsequently, P. Gahan demonstrated that *Escherichia coli* DNA carrying marker genes, which DNA does not contain the DNA insertion system found in *Agrobacterium tumefaciens*, could integrate into the plant host’s genome, modify it, and be expressed, showing a capacity for the horizontal transfer of DNA [178].

M. Stroun, P. Anker, and P. Gahan also developed the concept of transcession [171]: in repeating the experiments on bacteria, plants, and higher organisms, they identified a complex containing bacterial DNA and its DNA- and RNA-dependent DNA polymerases capable of integrating the bacteria genome into the host genome [19, 54, 179]. Upon the characterization and identification of a similar complex in higher organisms, these authors proposed the concept of virtosomes: a lipoprotein complex containing DNA, RNA, and the DNA- and RNA-dependent DNA polymerases [51]. This complex, playing the role of intercellular messenger, is found in the supernatant of culture medium and is actively secreted by cells in a regulated manner [20, 60] (see part 2.2.3).

However, it is not obvious that only virtosomes may play an intercellular messenger role. Given the apparent lack of a limiting membrane and their size which is comparable to small liposomes [180], it is likely that microvesicles might also be involved in transferring genetic materials from one cell to another.

#### Penetration into the cell

The uptake of virtosomes released from one cell type by a different cell type can result in a biological modification of the recipient cells. Anker, Stroun, and Gahan demonstrated in their works the transformation of NIH/3T3 cells on uptake of released mutant k-ras from SW480 cells [181], an allogenic T–B lymphocyte co-operation involving lymphocyte subsets from human donors with different allotypes [182–184] and DNA synthesis initiation in non-stimulated lymphocytes on uptake of virtosomes released by J774 and P497 tumor cells [60]. Thus, the virtosome appears to be a novel cytoplasmic component that may act as an intercellular messenger. While involvement of proteolipidonucleic acid complexes appears to have been demonstrated, nothing precludes that microvesicles could be able to show intercellular messenger function. A parallel with DNA complexes synthetically formed to transfer short DNA sequences such antisense or miRNA molecules, or longer DNA molecules such as plasmid DNA for regulating cell gene expression either *in vitro* or *in vivo* [180] buttress this notion.

#### Role in the cooperation between B lymphocytes and T lymphocytes in the humoral type of immune reaction

In 1975 and 1976, a series of experiments performed by M. Stroun, P. Anker, and D. Jachertz revealed that T lymphocytes, stimulated or not, released actively and spontaneously newly synthesized DNA, in a complex structure later identified as a virtosome, into their culture medium [60, 185, 186].

**Fig. 13:**
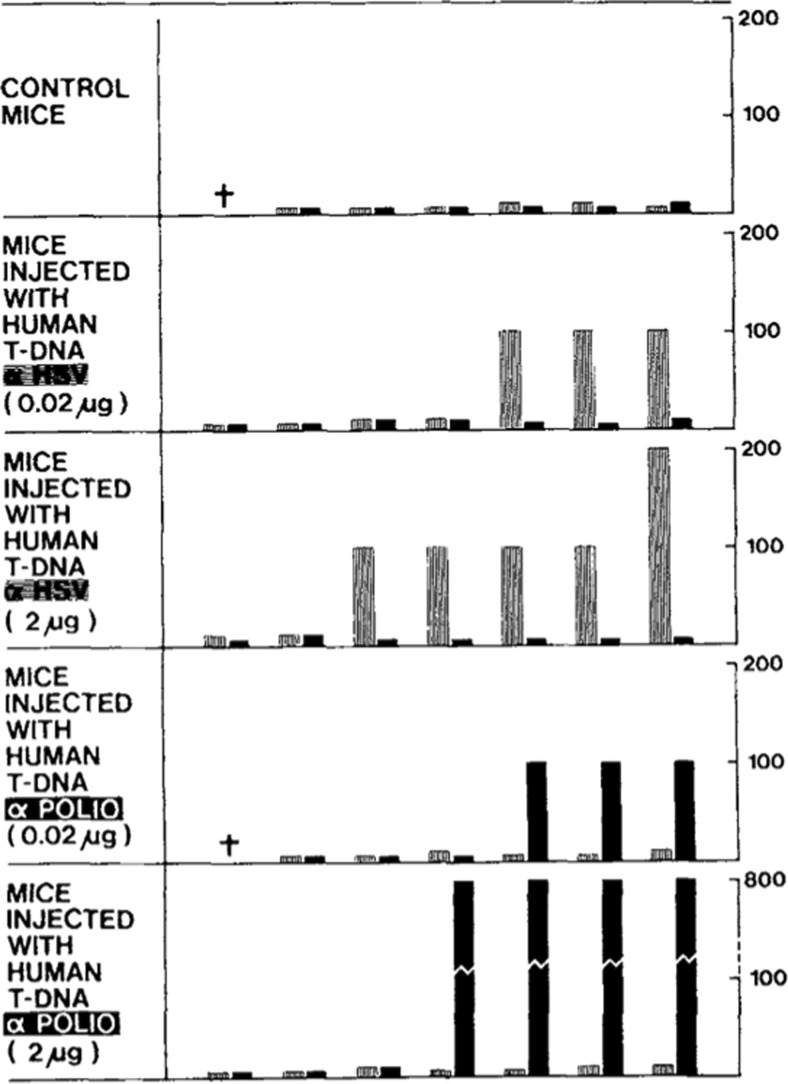
Representation of the main results showing the involvement of cirDNA released by T cells in the humoral immune response *in vivo*. Neutralizing activity of the serum of nude mice injected with DNA released by human T cells previously exposed to UV inactivated HSV or polio virus. Mice were injected with either 0.02 or 2 μg of human DNA. Control mice remained uninjected. After 5 days, the mice were killed and their serum was collected and frozen until tested. *Numbers in ordinate* represent highest dilutions of serum still presenting neutralizing activity (more than 3 units below virus control). Neutralization of HSV (*hatched area*); neutralization of polio (*solid area*). Human released T-DNA (α HSV) or T-DNA (α Polio) = DNA released by HSV or polio virus-exposed human T-lymph (from Anker et al. [184])

In 1979 and 1980, they conducted *in vitro* experiments on T cells and B cells from donors exposed to HSV [183, 187, 188]. Note that isolated T cells or B cells were not able to produce an anti-HSV response. However, when isolating the T cells’ released DNA following stimulation by HSV, and adding it to the B cells’ culture medium for 3 days, anti-HSV antibodies were synthesized with anti-allotypes of the T cell donor by the B cells. Furthermore, *in vivo* experiments have been conducted: nude mice were injected with DNA released by T cells exposed to HSV or polio viruses [184, 188]. Five days after injection, those nude mice, never previously exposed to pathogens, produced specific anti-HSV or anti-polio antibodies with human characteristics (Fig. 13). Those observations seem to indicate a genetic transfer of information between T cells and B cells: T cell released DNA could act as an intercellular messenger carrying the necessary information for B cells to code the corresponding antibody.

### Genometastasis

Among the physiological roles of cirDNA, the most obvious that one can imagine is the capacity of “transfection,” a theme that excites much interest among researchers. In 1965, Bendich et al. published in Science “Circulating DNA as a possible factor in oncogenesis” [189]. They demonstrated that tumor DNA injected into the mouse circulatory system was the origin of tumor development and proposed the hypothesis that DNA penetrated into healthy cells to render them tumorous. This observation has since been extensively confirmed and led to the concept of genometastasis [181, 190–192].

#### Discovery and concept

The genometastasis hypothesis is as follows: cirDNA released by tumor cells is capable of transfecting healthy cells at a distance from the primary tumor and leading to the formation of metastases [192].

**Fig. 14:**
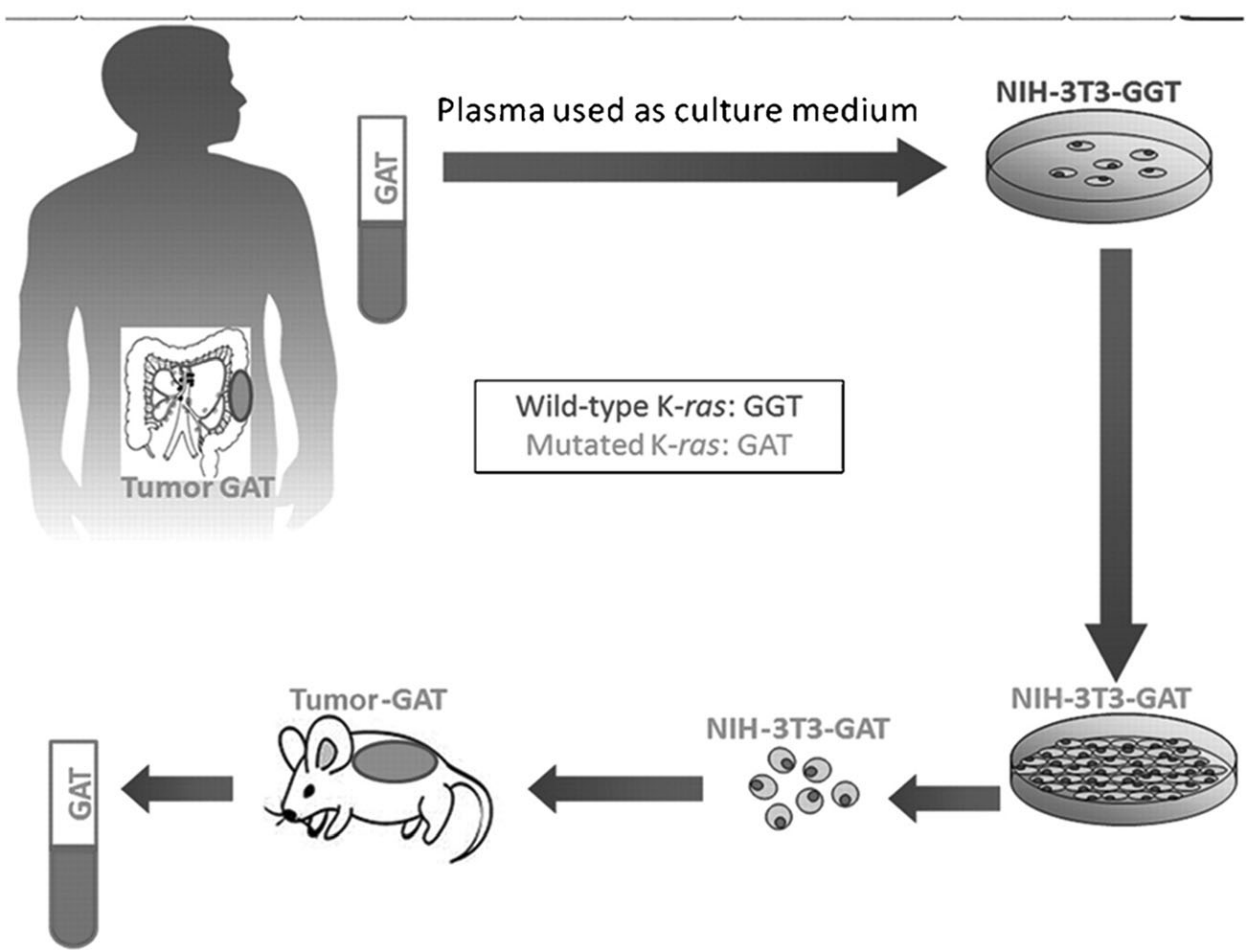
Schematic representation of the observing genometastasis. Plasma from colon cancer patients, in which a *KRAS* mutation had been detected, had been added to cultures of NIH/3T3 cells. Human *KRAS* DNA was detected soon afterward in these cells. Treated cells were injected, subsequently, into NOD-SCID mice and macroscopic tumors were generated. After sacrifice, human mutant *KRAS* sequences were detected in primary tumors, plasma, lungs, and livers of injected animals (from Garcia Olmo et al. [190])

P. Anker and his collaborators were the first to demonstrate the transfecting and transforming capacities of this extracellular DNA found in the culture medium of SW480 tumor cells on NIH/3T3 mouse fibroblasts [181]. After exposure to the SW480 culture medium, the NIH/3T3 cells not only became tumorous, but in addition, carried the *KRAS* mutation present in the SW480 cell line. In 2010, Garcia Olmo et al. observed the same event with plasma obtained from a colorectal cancer mutant *KRAS* carrier patient [190]. After adding this plasma to the culture medium, the NIH/3T3 cells became tumoral and carriers of the human *KRAS* mutation. Moreover, after the injection of these transformed NIH/3T3 cells into NOD-SCID mice, tumors containing the specific human *KRAS* mutation were developed (Fig. 14).

#### Hypothetical mechanisms

**Fig. 15:**
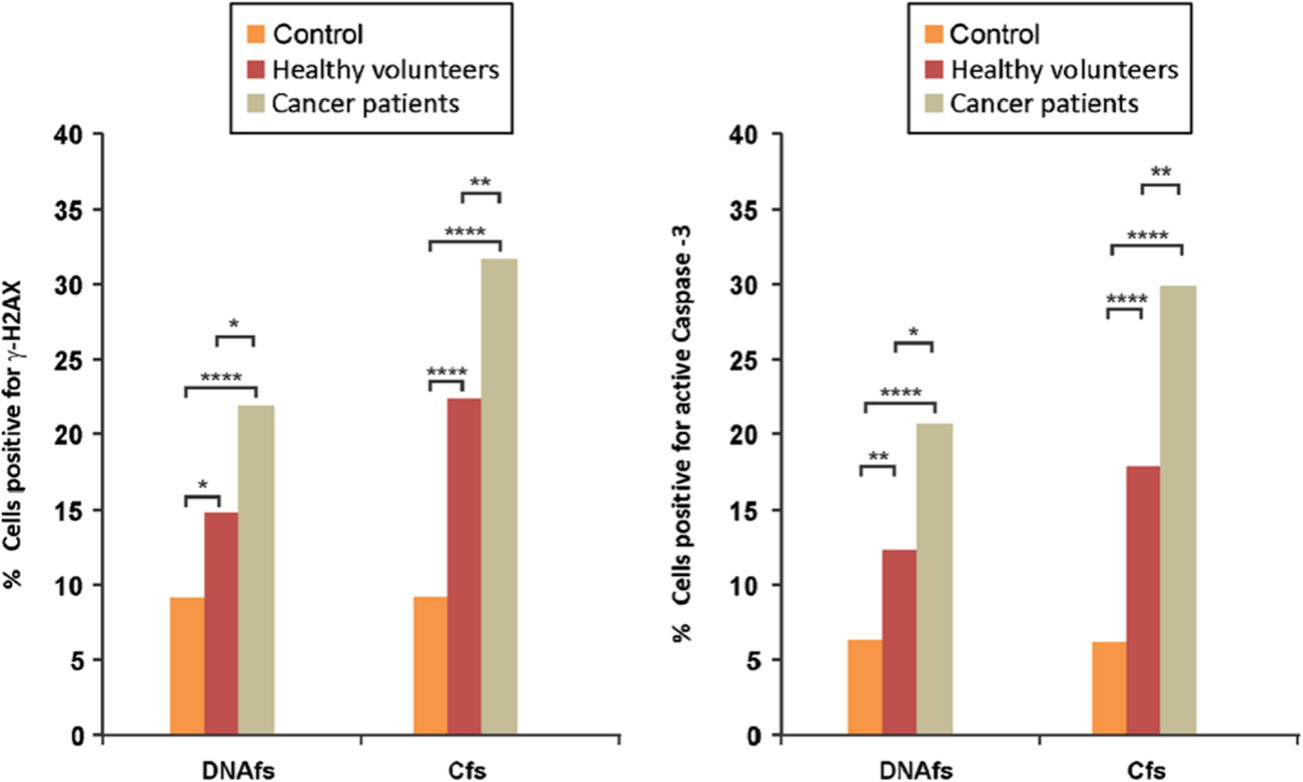
Induction of double-strand DNA breaks and apoptosis by cirDNA into NIH/3T3 cells. Induction of γ H2AX and active caspase-3 by fragmented circulating DNA (DNAfs) and circulating chromatin (Cfs) derived from healthy volunteers (*red bars*) and cancer patients (*gray bars*). *In vitro* analysis of γ H2AX and active caspase-3: NIH/3T3 cells (10 × 10^4^) were treated with DNAfs and Cfs (5 ng DNA each) for 6 h for detection of γ-H2AX (*left*) and for 24 h for detection of active caspase-3 (*right*) by immunofluorescence. For γ-H2AX (*left-hand panel*), 300 nuclei were counted and the percentage of nuclei showing positive foci were calculated and analyzed by chi-squared test. For active caspase-3 (*right-hand panel*), 200 cells were counted and the percentage of cells showing positive fluorescent signals were calculated and analyzed by chi-squared test (from Mittra et al. [195]) (color figure online)

Many structures carrying cirDNA can be involved in this genometastasis: it has been shown that apoptotic bodies can be responsible for this transformation at a distance, as can both microsomes and exosomes [193]. The complexing of cirDNA with proteins present in the circulatory system equally favors its cellular internalization. In addition, it has been shown that nucleosomes have the capacity to directly cross plasma membranes and to penetrate into the nucleus [194]. Virtosome could equally be involved in this phenomenon in penetrating other cells and modifying their genetic program [193]. Another recent study by Mittra et al. [195] clearly showed that there was an integration of cirDNA in host cells: after addition of human derived cirDNA to murine cells culture, they revealed the cellular entry, the nuclear uptake of human ccfDNA into murine cells and the colocalization of human ccfDNA and murine genomic DNA on karyotypes from metaphase spreads [195]. Furthermore, Mittra et al. showed that this uptake and genomic integration was responsible for apoptosis activation and DNA damage into host cells [196] (Fig. 15). Remarkably, those effects were greater with cirDNA from cancer patients as compared to that from healthy donors.

While those biological effects have been demonstrated, the mechanisms of DNA integration into the host genome need to be elucidated. Note that in 1969, Gahan, Anker, and Stroun reported that when roots of *Vicia faba* were treated with heterologous DNA (salmon sperm), DNA damage occurred in the host cells [197]. This appeared to be due to the heterologous DNA breakdown by DNAse on entry into the roots so increasing the nucleotide concentration in the cells and leading to chromosome damage. This did not occur with the homologous *V. faba* DNA [198].

Furthermore, membrane vesicles double-stranded DNA naturally found in the subcellular particulate fraction of some bacteria (such as Ruminococcus or Mycobacterium) are able of transformation [199]. It appears that membrane-associated heritable transformation plays a role in lateral gene transfer in complex microbial ecosystems. Acquisition of genetic information through horizontal gene transfer (HGT) is an important evolutionary process by which microorganisms gain novel phenotypic characteristics [200]. Several works support the idea that the genomes of certain eukaryotes have been exposed to exogenous DNA more frequently and continuously than previously thought [201].

In fact, horizontal gene transfer has happened between different *kingdoms* of life throughout the history of life on the planet [202, 203]. It seems that the genome of just about every modern species is something of a mosaic constructed with genes borrowed from many different forms of life. We must now acknowledge that, even among the most complex organisms, vertical is not the only direction in which genes travel [204]. Thus, HGT was prematurely revealed by early works from Anker and Stroun in plants or eukaryotic cells in mice in the way of the cirDNA discovery.

HGT is defined as the transfer of genes between organisms in a fashion other than is found through traditional reproduction. Thus, while in the case of the initial experiments defining genometastasis, the movement of the human mutant *KRAS* cirDNA to murine cells can be considered as a horizontal DNA transfer, the movement of self DNA within an organism cannot be also considered. We suggest also that other situations, whereby the cirDNA can enter cells and modify the biology of the recipient cells in the same organism, cannot be defined as HGT since the DNA is present as fragments of genes and not whole genes. We would suggest that this process is more correctly termed as intra-organism genetic transcession (IGT).

The term transcession was initially derived to describe the release of DNA by bacteria and its biological activity in plants and animal tissues [205, 206].

### Pro-inflammatory and immunological effects

#### Circulating DNA: a DAMP

##### Immunostimulatory characteristics of circulating DNA

DNA constitutes a macromolecule with immunostimulating properties. The particular DNA double-helix structure, the particular motifs of certain sequences and the molecular interactions are three factors at the origin of the stimulation of the immune response [207]. In effect, the exposure of cells of the innate immune system to double-stranded DNA notably provokes the activation of the genes regulating interferon secretion (INF) and other pro-inflammatory molecules. This stimulation is at the origin of a strong inflammatory response mediated by the secretion of cytokines. DNA alone can activate the immune system, but this can also occur on its complexing with other molecules [47, 207]. The abundant presence of nucleic acid receptors in the cell confirms its important role in the innate immune system [208]. In the normal state, it appears that the presence of DNAse in the circulatory system permits DNA degradation, blocking this stimulation. It has been shown that DNAse-deficient mice were particularly sensitive to the development of autoimmune pathologies [209, 210]. Twenty percent of LED patients are deficient in DNAse [211, 212].

##### Mechanisms of circulating DNA recognition

Some cirDNA recognition mechanisms have been demonstrated: one Toll-like receptor (TLR) 3-7-8-9 pathway and one independent TLR less important pathway that will not be detailed here [209]. The TLRs are implicated in the innate immune response and are present in dendritic cells and macrophages. It involves the receptors being implicated in recognizing particular molecular motifs derived from pathogenic organisms: the pathogen-associated motif pattern (PAMP) [213]. The binding ligand-TLR provokes the activation of transcription factors NFkB/AP1 that regulate the induced expression of inflammatory cytokines such as tumor necrosis factor (TNFα) or interleukin-1 (IL-1) or interleukin-6 (IL-6), and co-stimulating molecules CD80 and CD86. The molecular ligands can equally be of endogenous origin: fibrinogen, heat shock proteins, high-mobility group protein B1 (HMGB1), and self cirDNA. These endogenous ligands are called damage-associated motif pattern (DAMP) [209, 213, 214].

**Fig. 16:**
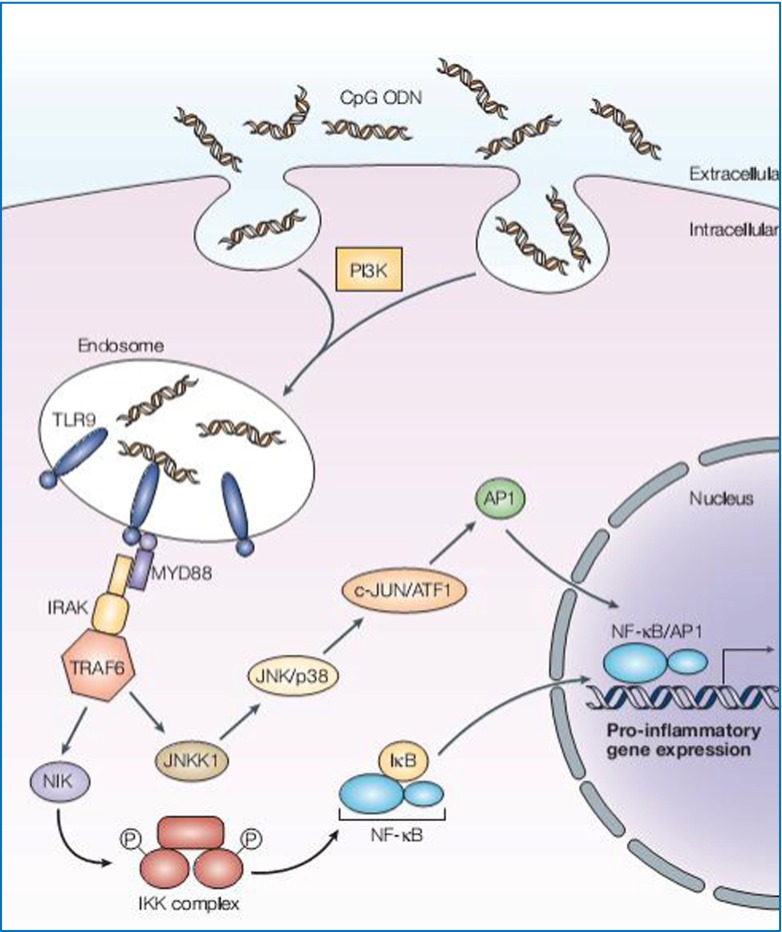
Interaction between DNA and TLR9. Class III phosphatidylinositol 3-kinase (PI3K) facilitates the internalization of CpG oligodeoxynucleotides (ODNs) into endosomal vesicles that contain Toll-like receptor 9 (TLR9). The interaction between CpG DNA and TLR9 transduces an intracytoplasmic activation signal. The signal initiates with the recruitment of myeloid differentiation primary response gene 88 (MYD88) to the Toll–interleukin-1 receptor (TIR) domain of TLR9, followed by activation of the IRAK–TRAF6 complex. This leads to the activation of both the mitogen-activated protein kinase (MAPK: JNK1/2 and p38) and inhibitor of nuclear factor-kB (NF-kB) kinase (IKK) complexes, culminating in the upregulation of transcription factors, including NF-kB and activating protein 1 (AP1). ATF1, activating transcription factor 1; IRAK, IL-1 receptor-activated kinase; JNKK1, c-JUN N-terminal kinase (JNK) kinase 1; NIK, NF-kB-inducing kinase; TRAF6, tumor-necrosis factor receptor-associated factor 6 (from Klinman [215])

In these particular pathological conditions, cirDNA is not degraded by the nucleases and thus can complex with other proteins and reach the cellular TLR implicated in nucleic acid recognition: TLRs 3, 7, 8, and 9. Among these receptors, TLR9, present in intracellular endosomal vesicles, but equally at the extracellular surface of polynuclear neutrophils (PNNs), is implicated in the recognition of small-sized oligonucleotides (20–30 nt) containing demethylated CpG motifs. The binding of these ligands by TLR9 is at the origin of an innate immune response characterized by an inflammation mediated by the secretion of cytokines by the action of NF-kB (Fig. 16) [215].

#### The particular case of circulating mitochondrial DNA

**Fig. 17:**
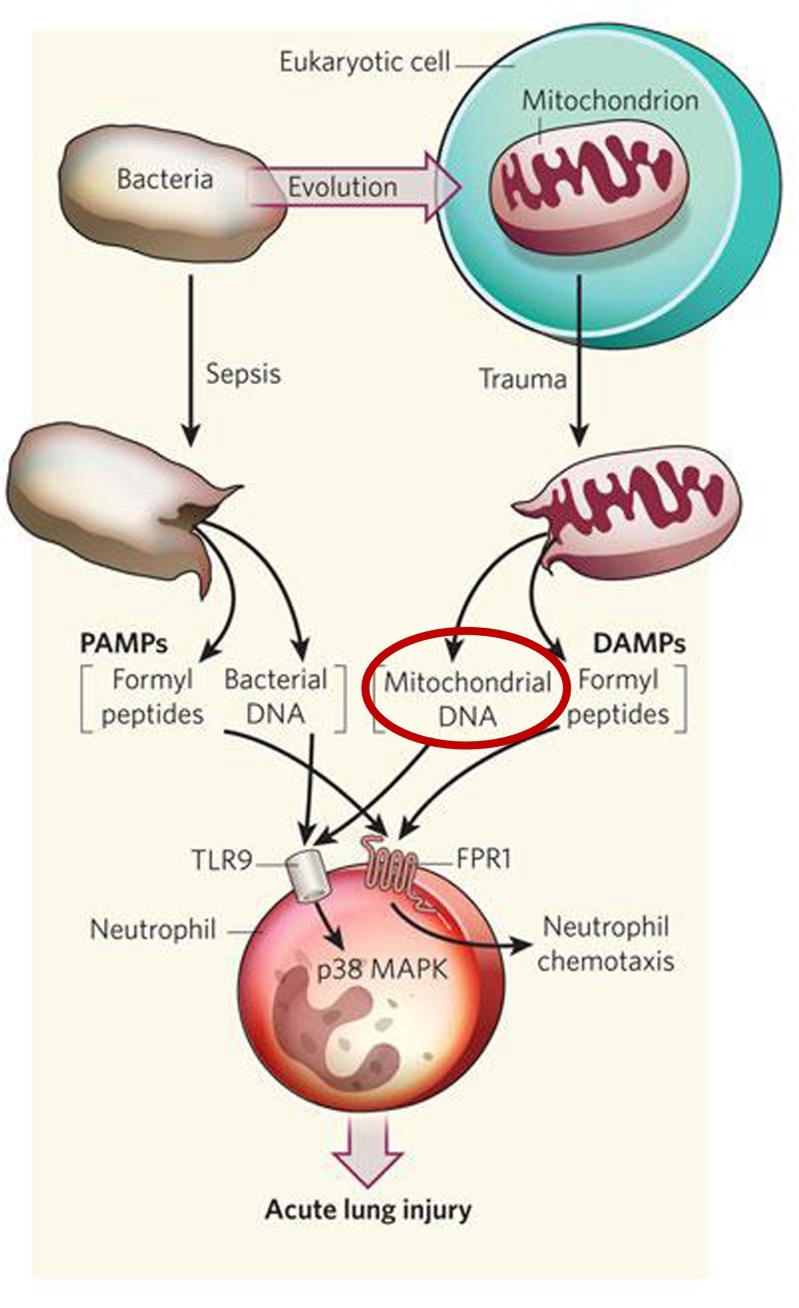
Involvement of mitochondrial cirDNA in the inflammatory process. Similar to the release of bacterial DNA following sepsis, the mitochondrial DNA released by severe trauma can also act through the Toll-like receptor 9 (TLR9) to activate neutrophils by activating p38 MAP kinase (MAPK). *DAMPs* damage-associated molecular patterns, *PAMPs* pathogen-associated molecular patterns. Adapted from © 2010 Nature Publishing Group Calfee, C. S. & Matthay, M. A. Clinical immunology: culprits with evolutionary ties. Nature 464, 41–42 (2010). All rights reserved

Due to the bacterial origin of mitochondria [216], circulating mitochondrial DNA presents a particular structure able to activate the immune system because of the similar structure between mitochondrial and bacterial DNA [98]. In effect, mitochondrial DNA presents the same circular structure as bacterial DNA, is protein-free, and possesses a number of demethylated CpG islands. Its release under certain pathological conditions can lead to the activation of the immune system and an unleashing of an inflammatory response. Some works have shown that the mitochondrial DNA activates the polynuclear neutrophils on specific binding to TLR9 [217] (Fig. 17). This interaction is at the origin of the inflammatory response via p38 [92].

#### Clinical manifestations

The first evidence of the immunostimulatory properties of cirDNA dates from the 1960s and the studies of SLE [27]. One characteristic of such patients is the presence of anti-cirDNA antibodies. cirDNA is, thus, characterized as an antigen and the immune complex anti-cirDNA antibodies—cirDNA attaches to the basal membranes to provoke an inflammatory reaction [218]. Subsequently, cirDNA analysis in states of inflammation and autoimmune pathologies constitutes a vast fundamental and clinical research field [32]. A relationship between increased cirDNA of fetal origin in women with a complicated pregnancy and some cases of either spontaneous abortion or premature births has been demonstrated [219]. The very high concentration of fetal cirDNA found with some complications such as pre-eclampsia can be explained by an abnormal level of cell death of fetal cells [220, 221]. The fetal DNA being hypomethylated has been shown to bind to TLR9 receptors of the mother’s immune cells so unleashing an inflammatory response leading to the situations of either premature birth or spontaneous abortion [219].

### Other mechanisms involved in tumor progression mediated by circulating DNA

The identification in 2004 of new structures such as the “neutrophil extracellular DNA traps” (NETS) [142] and later the “eosinophil extracellular DNA traps” (EETS) [153], and their mechanism of liberation by ETosis, opens a particularly appealing new field of investigation. These extracellular DNA structures are secreted by “polynuclear” cells following their stimulation by either pathogenic agents or under certain physiopathological conditions such as inflammation [222]. The involvement of these structures in processes such as hypercoagulation or autoimmune pathologies have been described and, interestingly, high concentrations of cirDNA have been well-documented for patients presenting with pathophysiological problems [223] (see Sect. 2.2.3). The NETs could be implicated in tumor progression [224]. It has already been described that high levels of circulating polynuclear neutrophils are positively correlated with a reduction of the global survival rate because they may be responsible for metastatic progression [225]. Thus, it has been shown that the polynuclear neutrophils intervene as “bridges” between the circulating tumor cells and the hepatic and lung surface epithelia, favouring their adhesion to the surface of these organs and *in fine* metastatic dissemination. Equally, they could secrete factors aiding the adhesion of the circulating tumor cells to endothelial surfaces [226]. Recent work has shown links between NETs, circulating tumor cells and metastatic progression using a mouse lung cancer model [101]. In this study, the authors demonstrate that the NETs capture circulating tumor cells in the blood, aiding their adhesion to hepatic endothelia and hence metastatic dissemination. This observation shows the “anchorage” capacity of the NETs and their possible roles in migration and adhesion. Interestingly, it has been shown that the EETs contain only mitochondrial DNA, the absence of nuclear DNA indicating an active mitochondrial DNA release in certain conditions [154].

### A potential therapeutic target

**Fig. 18:**
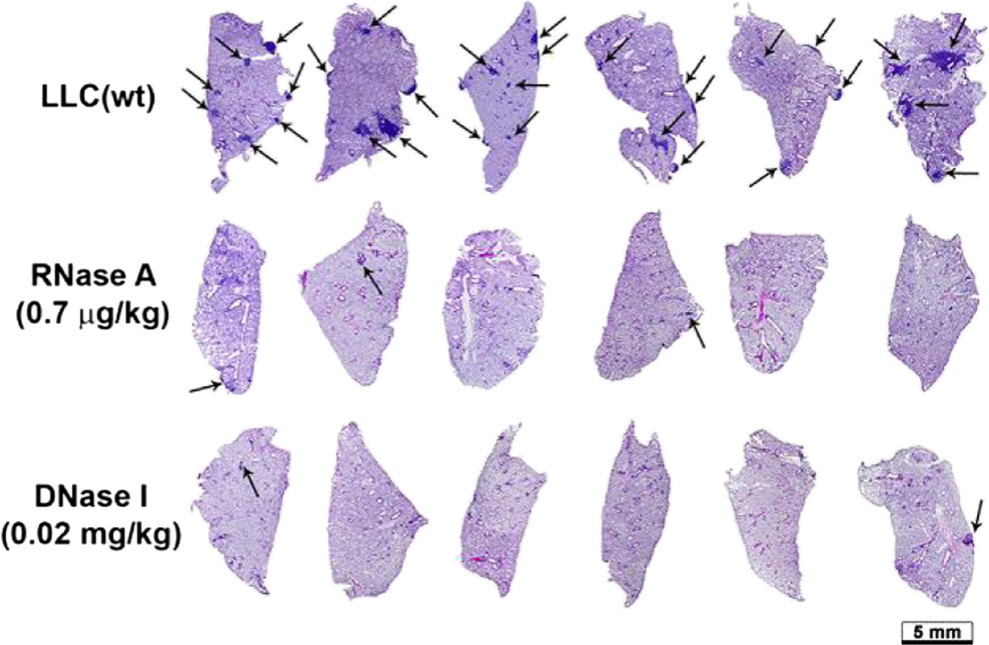
The metastasis suppression effect of RNase A or DNase I on Lewis lung carcinoma (LLC) metastatic mouse model. Typical histotopograms of lung lobes in the control and experimental groups treated with RNase A and DNase I. Hematoxylin and eosin staining. *Arrows* indicate large metastases. *Bar* corresponds to 5 mm (from Patutina et al. [228])

The description of the biological and the pathophysiological effects described in this review raises the obvious question of considering cirDNA as a therapeutic target. This concerns two major principles namely, the implication of cirDNA in tumor progression via genometastasis and its role as a danger signal activating the immune system. Some researchers have considered that treatments by DNAse/RNAse, via a general pathway, permitting the destruction of these cirDNAs so as to reduce tumor progression [227]. Various cancer cell lines have been xenografted onto mice to test the efficacy of DNase treatment. For instance, experiments have been performed *in vivo* on lung tumor-bearing mice and have shown that the daily administration of DNase was at the origin of the reduction of metastatic progression [228, 229] (Fig. 18). Some preliminary experiments performed *in vivo* on rats that have developed tumors following injection of extracellular DNA derived from the culture medium of SW480 colorectal carcinoma cells by the phenomenon of genometastasis have shown that treatment with DNase was responsible for the reduction of tumor progression [230]. DNase or neuro-elastase inhibitor treatment should also be effective in the destruction of NETs implicated in metastatic spreading by sequestration of CTCs [101]. Another group was interested in the general administration of cationic polymers that permitted a complexing with cirDNA by electrostatic interactions [231].

Their *in vitro* and *in vivo* results have shown that certain polymer formulations permit inhibition of the inflammatory reaction while maintaining a normal activity of the immune system with respect to other danger signals such as viruses [232].

These original studies shed a light upon an aspect, currently little documented, on cirDNA and could open up interesting areas in the field of cancer, inflammatory, and autoimmune therapeutics.

## Circulating DNA: from controversy to breakthrough in oncology

The hypothesis of cirDNA originated from basic research observations made from plant graft hybrid experiments performed by Maurice Stroun following on from the work of the Russian biologists of the 1950s. He made this hypothesis to explain why, in his graft experiments made on eggplants, some characteristics of the mentor plant appeared in the grafted pupil plant. The concept of cirDNA was, of course, not very easy to impose and the fact that it originated from not always controlled experiments did not help. Maurice Stroun’s data, once published in 1962 [16, 176], were hardly believed, and, as a consequence, he lost his funding from the Swiss National Fund for Scientific Research [233]; he could not even have access to the criticisms of the experts. Professor Chodat, Director of the team where he worked, in particular the works in collaboration with Sidransky, backed him but, consequently lost the possibility to become Rector of the University of Geneva [233].

The graft hybrid experiments were considered to be a danger for the university and even for the reputation of Switzerland. Following months-long struggles with the university, Maurice Stroun resigned his post and left Geneva where he had no opportunity to work. After a few years in Belgium and in Israel, he came back in 1970 to Geneva where he worked with Philippe Anker. Severe criticisms occurred when they published that living cells, frog auricles, and human lymphocytes spontaneously released DNA [170, 187]. The difficulties and pressures again returned from both the university and the Swiss National Fund for Scientific Research. In spite of the publication by J.C. Rogers in 1972 [52] of a report showing that stimulated lymphocytes released newly synthesized DNA, the Swiss National Fund refused to grant a research project because some experts postulated that either the discovered molecule was not DNA or that the observation was due to contaminating bacteria. Grant applications from Drs. Stroun and Anker were refused on each occasion, and some critics were straightforward without any explanation using comments such as “miles away from real Science.” Following several refusals and even falsification of the expert comments of the reviewers on a grant application to make them negative, Maurice Stroun and Philippe Anker undertook a libel suit for defamation in 1985, but the judge of the Department of Justice of the canton of Bern abandoned the affair. While in 1987 and 1989, Maurice Stroun and Philippe Anker had reached some international fame with the discovery of circulating nucleic acids in the plasma of cancer patients [22, 46], had published in top journals, in particular works in collaboration with Sidransky, and had been invited all over the world, grant applications continued to be obstructed. Despite, finally, some funding from the Swiss League against Cancer, more money was needed for performing further investigations. Without help from some private donors, they would have had to close their laboratory.

The fields of science are full of crucial discoveries that were not taken into consideration at time of their reporting. Rarely, the discovery of a class of biological compounds was so retarded in their applications as was cirDNA. Since their discovery in 1948 it took 57 years before starting to clinically evaluating their potential. In the light of the various clinical applications, especially in oncology, now in progress such as diagnosis, prognosis, therapy monitoring, or patient follow-up, the forces impeding full recognition of this discovery should be analyzed by the scientific community so as to overcome any future, similar difficulty.

The early obstinate position of first Drs. Stroun and Anker illustrates that human resilience is sometimes a requisite for an emerging discovery. However, sometimes this resilience may be confronted by a scientific community that is inclined to maintain existing paradigms or dogmas. It appears that late recognition of P. Anker, M. Stroun, and P. Gahan was damaging to cancer science and patients. It is certain that cirDNA is a breakthrough discovery that would impact standard management care of cancer patients as well as other clinical disorders [35]. For instance, works from the groups of L. Diaz [67, 169], N. Rosenfeld [75, 234], A. Bardelli [235], AR Thierry [70, 74], or N. Turner [236] recently demonstrated the clinical validation and utility of cirDNA in oncology.

**Fig. 19:**
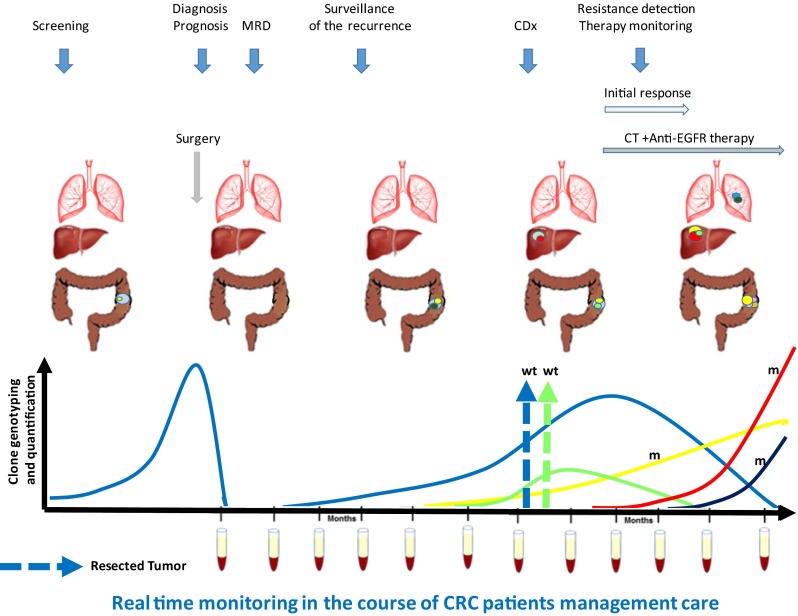
Real-time monitoring in the course of colorectal patients management care by cirDNA analysis. *m* mutant, *WT* wild type, *MRD* minimal residual disease, *CDx* companion diagnostics, *CT* chemotherapy, *CRC* colorectal cancer

Following the first demonstration of the capacity of detecting *RAS* mutations from cirDNA analysis by Vasioukhin et al. [23] and Sorenson et al. [24], in collaboration with P. Anker and M. Stroun, in acute myeloplastic syndrome and pancreatic cancer, respectively, Diehl et al. presented the first clinical evaluation of the detection of molecular alterations from cirDNA [26]. Thierry et al. reported in 2014 the first clinical validation of the use of the cirDNA analysis in oncology, by examining the *KRAS/BRAF* mutations in colorectal cancer patients [70]. CirDNA was shown to help in dynamically estimating the tumor burden and to follow-up patients during management care (Fig. 19) [169, 234, 237]. Non-invasive analysis of acquired resistance to cancer therapy by analyzing plasma DNA was later demonstrated in breast and colorectal patients [75, 235, 238]. Lastly, Garcia-Murillas et al. [236] showed that quantitative mutation tracking in serial samples increased sensitivity for the prediction of relapse in early breast cancer suggesting an application of cirDNA in the surveillance of the recurrence and detection of the minimal residual disease. Thus, qualitative as well as the quantitative analysis of cirDNA in various malignancies are now intensively investigated and critical data are expected to be exponentially reported toward probable changes in the cancer clinical practice (see review [33, 34]). In addition to the predictive information and perhaps screening capacities as determined from tumor tissue analysis, tracking resistance, surveillance of the recurrence, detection of the minimal residual disease or screening are solely enabled by analyzing cirDNA facilitating longitudinal personalized medicine for cancer patients (Fig. 19). A most recent evidence for the development of cirDNA is the positive approval emitted by the Committee for Medicinal Products for Human Use (CHMP) of the European Medicines Agency (EMA) for Iressa® (gefitinib). This allows doctors to identify suitable lung cancer patients by testing for *EGFR* mutations that are predictive of therapeutic response with this drug in the absence of a tumor sample. As the clinical utility of this application appears straightforward, this would pave the way to a larger utilization of cirDNA in oncology practice. Thus, if predictive information (companion test) and monitoring drug resistance are the two first approved clinical applications, it is highly probable that the future applications will be on monitoring treatment therapy, prognosis, minimal residual disease, surveillance of the recurrence/noninvasive follow-up of the disease, or even a screening test (Fig. 19).

**Fig. 20:**
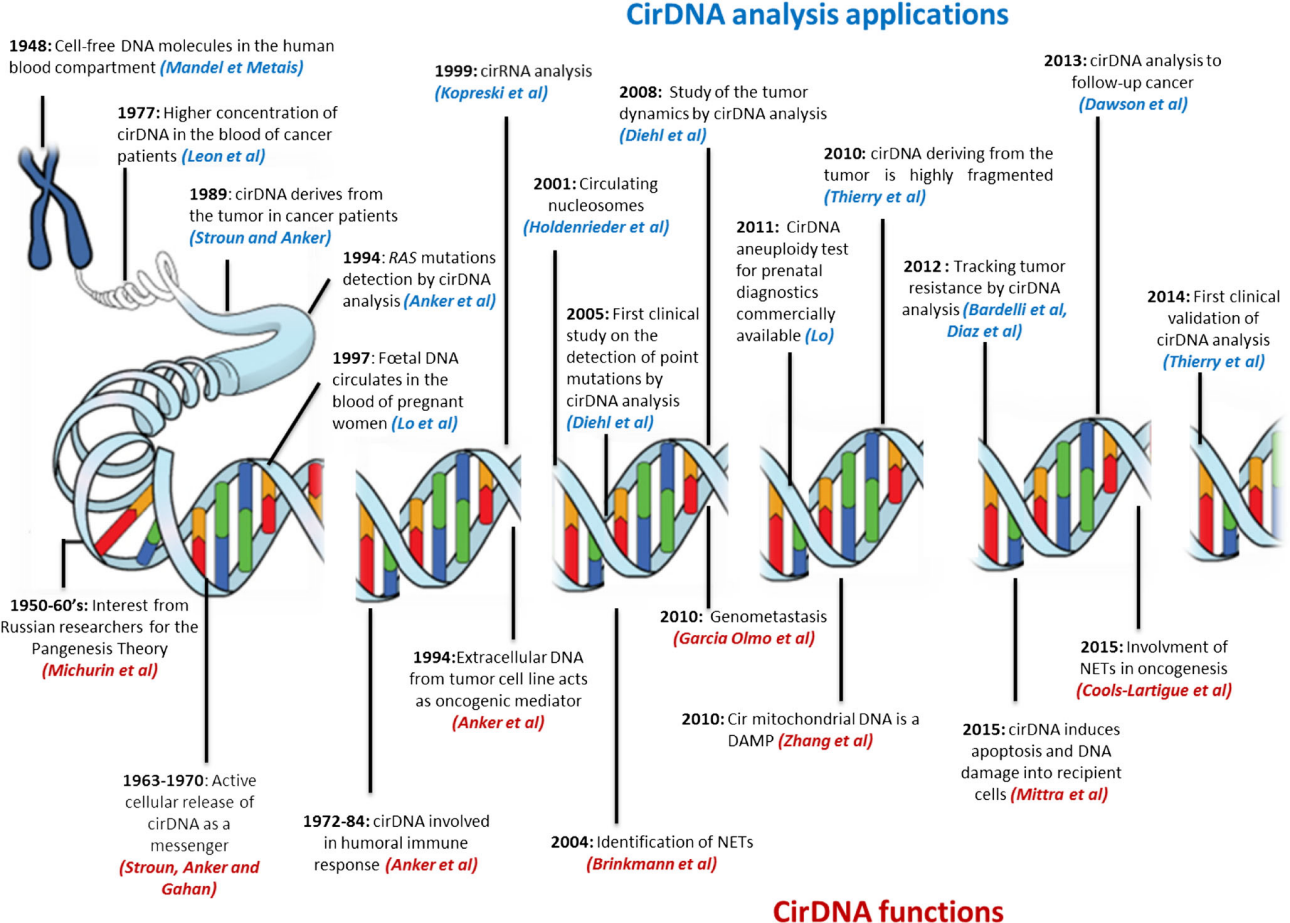
Timeline of the main important discoveries about circulating DNA applications and functions

This field benefited from development of technologies such as Q-PCR-based methods (e.g., allele-specific Q-PCR, digital Q-PCR, BEAMing), sequencing-based methods (e.g., next-generation sequencing, Cap-Seq, TamSeq), or chromatographic-based methods such as advanced mass spectrometry [34]. Their further development or the emergence of new, advanced technologies would allow for highly specific analysis of cirDNA. In association with the better understanding of the structural properties of cirDNA, both biological and functional (Figs. 11 and 20), with a focus on the tumor and metastasis processes, such future progress would warrant wide cirDNA clinical applications in oncology.
